# Addressing multiple bit/symbol errors in DRAM subsystem

**DOI:** 10.7717/peerj-cs.359

**Published:** 2021-02-09

**Authors:** Ravikiran Yeleswarapu, Arun K. Somani

**Affiliations:** Department of Electrical and Computer Engineering, Iowa State University, Ames, IA, USA

**Keywords:** DRAM reliability, Reed solomon code, Chipkill, Hash, Silent data corruption, Multiple bit errors

## Abstract

As DRAM technology continues to evolve towards smaller feature sizes and increased densities, faults in DRAM subsystem are becoming more severe. Current servers mostly use CHIPKILL based schemes to tolerate up-to one/two symbol errors per DRAM beat. Such schemes may not detect multiple symbol errors arising due to faults in multiple devices and/or data-bus, address bus. In this article, we introduce Single Symbol Correction Multiple Symbol Detection (SSCMSD)—a novel error handling scheme to correct single-symbol errors and detect multi-symbol errors. Our scheme makes use of a hash in combination with Error Correcting Code (ECC) to avoid silent data corruptions (SDCs).

We develop a novel scheme that deploys 32-bit CRC along with Reed-Solomon code to implement SSCMSD for a ×4 based DDR4 system. Simulation based experiments show that our scheme effectively guards against device, data-bus and address-bus errors only limited by the aliasing probability of the hash. Our novel design enabled us to achieve this without introducing additional READ latency. We need 19 chips per rank, 76 data bus-lines and additional hash-logic at the memory controller.

## Introduction

Failures in DRAM subsystem are one of the major sources of crashes due to hardware errors in computing systems (Reliability data sets, 2005). As DRAM technology continues to evolve towards smaller feature sizes and increased densities, faults in DRAM devices are predicted to be more severe. For example, field study at Facebook (Meza et al., 2015) indicates that recent DRAM technologies have higher failure rates, up to 1.8×, when compared to previous generation.

Small cell dimensions limit the charge that can be stored in them. This results in lower noise margins. As cell density increases, coupling (or crosstalk) effects come into picture. In-fact, researchers have recently identified “disturbance error” (Kim et al., 2014) in newer DRAM devices. This error has a cross-device correlation, hence will lead to multi-bit errors across different devices in a rank.

Each generation of DDRx family has doubled the transfer rates and reduced I/O voltages, and therefore, transmission errors in the Memory controller-DIMM interface are on the rise (Meza et al., 2015; Jacob, Ng & Wang, 2007; Kim et al., 2016). Errors in data-bus along with growing device failures increase the frequency of multi-bit errors in the data fetched from DRAM subsystems. Address bits are also prone to transmission errors. Errors in address bus during READs lead to Silent Data Corruptions with current CHIPKILL designs. During WRITEs, errors in address bits lead to unrecoverable data corruptions. Field study (Sridharan et al., 2015) reports that “Command Address Parity (JEDEC, 2020a),” is necessary to tolerate the address errors in current servers. In this work, we also describe how faults in address bus lead to multiple symbol/bit errors. Given these trends, solely relying on circuit based schemes to protect against these transmission errors is not power-efficient (Kim et al., 2016).

Field studies from Siddiqua et al. (2013) and Meza et al. (2015) describe their findings on channel faults. Specifically, Meza et al. (2015) states that channels generate a large percentage of errors when they fail—they contribute to 21.2% of all errors each month, but they occur only in a small fraction of failed servers (about 1.10%). It also states that these channel faults can be either transient—due to misalignment of transmission signals on the channel or permanent—due to failures in channel transmission logic on the DIMMs. These channel faults result in multiple bit/symbol errors across cacheline stored in a rank of DRAM devices in DIMMs.

Most servers use CHIPKILL (Dell, 1997) based reliability schemes, which can tolerate one or two symbol errors per beat. Multiple bit errors spread across the chip boundaries of a rank may not be detected by these schemes. Numerous field studies such as Meza et al. (2015), Sridharan et al. (2015), Siddiqua et al. (2013) and Schroeder, Pinheiro & Weber (2009) studied large scale data-centers and predict that future exascale systems will require stronger reliability schemes than CHIPKILL. These studies base their analysis using limited protection mechanisms/logging capabilities and, therefore, the actual failure rates might be greater than their assessments.

The industry is also realizing the need to effectively tolerate greater than one symbol errors. For example, in 2012, IBM introduced RAIM—Redundant array of independent memory (Meaney et al., 2012) technology to tolerate channel faults along with full DIMM and DRAM failures for mainframes. Based on the above observations in the research community and in the industry, in this work, we address the important problem of multi-bit/symbol errors in DRAM subsystem.

We first describe our error model, which captures the effects of various type of faults that may occur in DRAM devices, data-bus and address bus. This model complements recent efforts such as AIECC (Kim et al., 2016), which focus on faults in address, command and control signals. We then propose a new error handling mechanism—Single Symbol Correction Multiple Symbol Detection (SSCMSD) (Yeleswarapu & Somani, 2018). As single symbol errors/beat are more frequent (Sridharan et al., 2015), our mechanism uses ECC to correct them. In addition, we use a hash function to detect the less frequently occurring multi-bit (or symbol) errors. A hash function will detect multi-symbol errors with a high probability. It is the judicious combination of the two, that is, ECC and hash that makes our scheme effective.

During memory WRITE operation, we use a non-cryptography hash function to generate a checksum of the cache line’s data and address. We then encode this checksum along with the cache-line data using Single Symbol Correct (SSC) encoder and store the encoded data and checksum in the DRAM. During READ, we use the SSC-decoder to retrieve data and checksum from the stored codewords. If the decoder detects single symbol error in a codeword, it accordingly corrects it. The SSC-decoder cannot detect presence of multi-symbol errors all the time. We therefore recompute hash of the retrieved data and compare it with retrieved checksum. If these checksums match, with a high probability there is no corruption in the fetched data. When they do not match, multiple symbol data errors must have occurred. Our simulations show that SSCMSD provides protection against DRAM device errors and also acts as a busguard (as it enhances detection capability against data and address-bus errors).

We believe that SSCMSD is a very effective reliability mechanism for HPC/data-centers. More frequently occurring single symbol errors are corrected to achieve low recovery time. On the other hand, relatively infrequent, multi-symbol errors are detected by SSCMSD.

Our mechanism supports Selective Error Protection (SEP) (Mehrara & Austin, 2008) as the OS can selectively enable/disable the enhanced detection capability for different memory pages. It also supports retirement mechanisms such as Redundant bit steering (RBS) (Blackmon et al., 2003). With RBS, if one chip/bus has a permanent fault, the OS can scale back the reliability from SSCMSD to CHIPKILL by disabling the hash and use the redundant chip intended for the hash as a replacement for the faulty chip/bus.

The article is organized as follows. “DDRx Memory Organization” introduces DDRx subsystem. “Prior Work” describes prior work in the area of memory reliability. In “Preliminary Motivational Experiments”, we describe our preliminary experiments to understand the performance of Single Symbol Correcting Reed Solomon (SSC-RS) code in the presence of multi-symbol errors. Our error model is described in “Error Model”. “SSCMSD—A Novel Architectural Solution For Multi-bit/Multiple Symbol Errors” details the SSCMSD scheme. In “Evaluation”, we evaluate our scheme for its capabilities. In “Hash Selection”, we compare the properties of different hash functions suitable for SSCMSD design. “Tradeoff Analysis With Baseline Scheme” compares the costs/tradeoffs of SSCMSD scheme with the baseline. In “Conclusion”, we summarize our conclusions.

## DDRx Memory Organization

**Figure 1:**
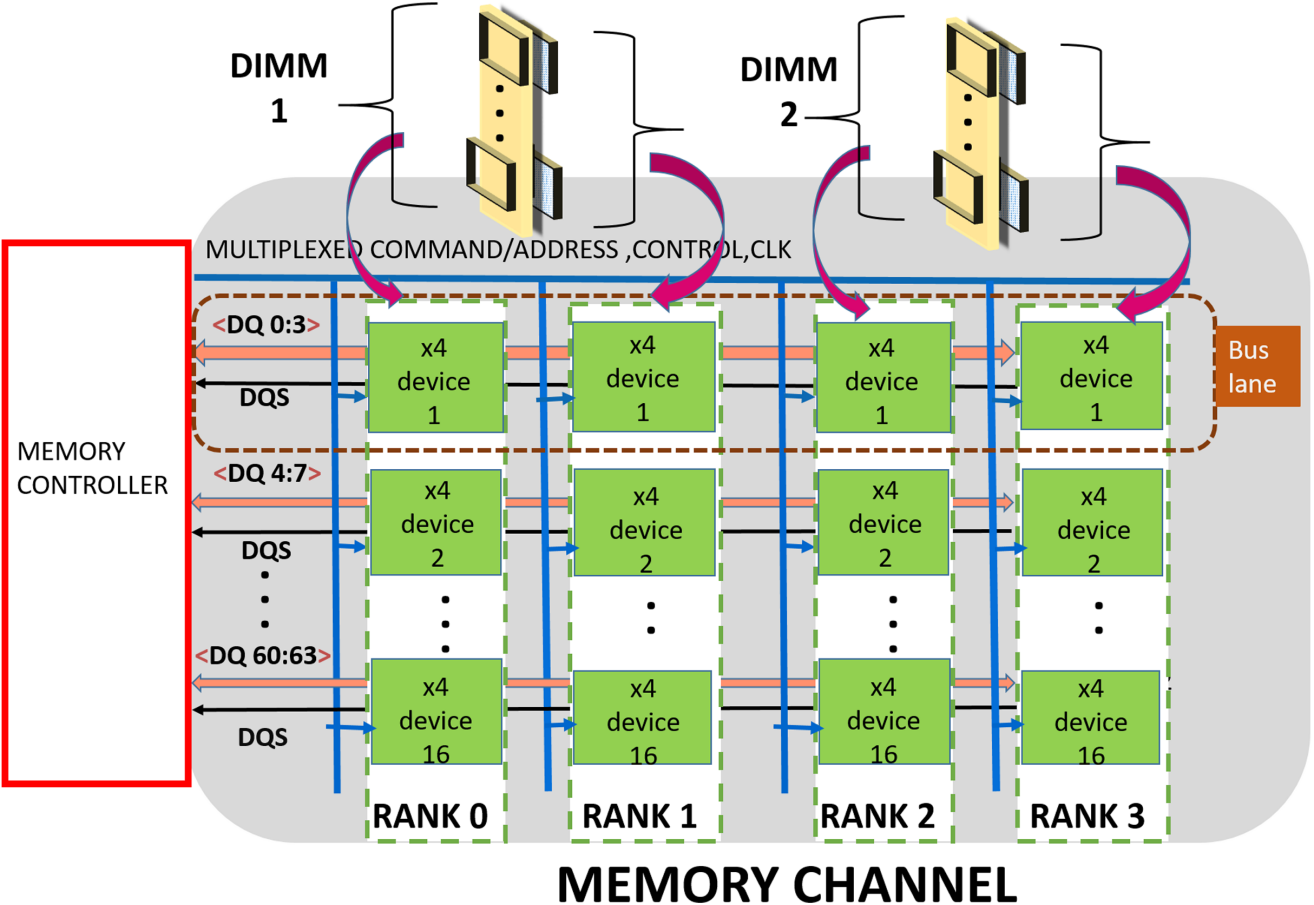
Memory channel—Memory controller is connected to DRAM modules (DIMMs) through shared bus.

A DDRx (Jacob, 2009; Kotra et al., 2017) based memory is organized into hierarchical groups to enable designers to trade bandwidth, power, cost and latency while designing memory subsystems. At the topmost level, the subsystem comprises one or more channels. Each channel is made up of one or more DDRx DIMMs, a shared Data-bus, Clock, Control and Command/Address (CCCA) bus signals. Each DIMM includes multiple DRAM chips which are grouped into multiple “ranks”. Typically, each DIMM has one, two, four or eight ranks. Furthermore, each chip has multiple independent banks. Each bank is composed of multiple sub-arrays (Kim et al., 2012) and a global sense amplifier. Each sub-array is further organized into a matrix of rows and columns with a sense amplifier. Figure 1 shows the organization of a channel which is composed of two ×4 (transfer width—4 bits) based DIMMs.

The data bus is organized into sixteen groups or “lanes”, each lane is composed of 4 bit-lines and is shared by devices (or chips) across a channel. The CCCA signals drive all the devices in the channel and hence operate at lower (typically, half of data) frequencies for meeting timing and reliability requirements. DDR4 (JEDEC, 2020a) has a 28 bit (22-Address/Command, 4 Control, 1 Clock) wide CCCA bus.

The memory controller (MC) handles memory requests from processor/cache/IO devices. As shown in Fig. 1, the MC communicates address, commands, and data to the DRAM ranks over the channels. Typically, read/write cache miss require 64-byte data to be transferred between MC and DRAM memory subsystem. In this article, we refer this 64-byte data (plus additional redundancy if any) as a cache-line. This is communicated in eight “beats” (8-half bus cycles). For a DRAM subsystem composed of DDR4, ×4 devices, each beat activates an entire rank (Tang et al., 2016) (16 devices) and MC fetches/sends 64 bits of data per beat. The bits (4 for ×4 devices) contributed by each device per beat are commonly referred as a word.

DRAM devices are currently available in ×4, ×8 and ×16 variants. A total of ×4 DDRx DRAMs are used widely in servers as they provide higher channel capacity at the cost of more energy. In this work, we present a robust ECC mechanism for ×4 based DRAM subsystem. Therefore, our solution will be suitable for servers where reliability is an important requirement.

## Prior Work

This section summarizes schemes currently used by the industry and recent academic efforts to improve the reliability of DRAM subsystem. SECDED (Jacob, Ng & Wang, 2007) and CHIPKILL (Dell, 1997) mechanisms were developed to address DRAM device errors. JEDEC introduced four schemes in DDR4 [5], to partially address signal integrity errors. MEMGUARD (Chen & Zhang, 2014), Bamboo-ECC (Kim, Sullivan & Erez, 2015) and AIECC (Kim et al., 2016) are recent academic efforts which are closely related to our work.

### SECDED

In 1990s, memory modules in servers were protected by using SECDED Codes. These codes make use of redundant (or check) bits to correct single-bit or detect double bit errors in a beat. For a typical beat size of 64 bits, SECDED code (Jacob, Ng & Wang, 2007) makes use of eight redundant bits. SECDED design can correct 1-bit error or detect 2-bit errors in 64 bits (per beat) with 12.5% redundancy and 8 additional bus lines/channel. In practice, it can detect/mis-correct some multi-bit errors (Kim, Sullivan & Erez, 2015) as well.

### CHIPKILL correct

As the demand for larger, high-density memory modules increased in the server industry, there was a need to protect against a single device failure. IBM introduced the “CHIPKILL Correct” error model to tolerate the failure of a single DRAM device in a rank.

CHIPKILL implementations make use of Reed Solomon (RS) Codes. RS codes use Galois “symbol” (set of bits) based arithmetic (Geisel, 1990) and like SECDED use additional logic to generate codewords (set of data and check symbols) using data symbols. The circuit complexity of RS code increases with the symbol size. Therefore, small symbol sized RS codes such as 4-bit and 8-bit are more commonly used. There are two popular versions of chipkill.

#### 4 check symbol based SSCDSD (Single symbol correct, double symbol detect) CHIPKILL

AMD’s 2007 design (AMD Inc, 2007) and Sun UltraSPARC (Sun Microsystems, 2008) provide SSCDSD capability for ×4 DRAM devices by using 4-bit symbol RS code with four check symbols. To maintain redundancy at 12.5%, this design uses 32 data symbols (128 bits), 4 check symbols (16 bits) per beat with 144-bit data bus and 36 devices per rank. The scheme by design “over fetches”, that is, two cache lines are accessed during a memory transaction (8 beats * 32 data devices/rank * 4 bits = 128 Bytes) and uses 144 bit data bus. Therefore, it may result in increased energy consumption.

#### SSC (Single symbol correction) CHIPKILL

To reduce access granularity to one cache-line, in 2013, AMD developed a Single Symbol Correction based 8-bit symbol RS code (AMD Inc, 2013) for ×4 DRAM devices. Similar to organization for SECDED, this scheme also uses 72 bit data bus and 18 devices (16 data and two for storing check symbols) per rank.

**Figure 2:**
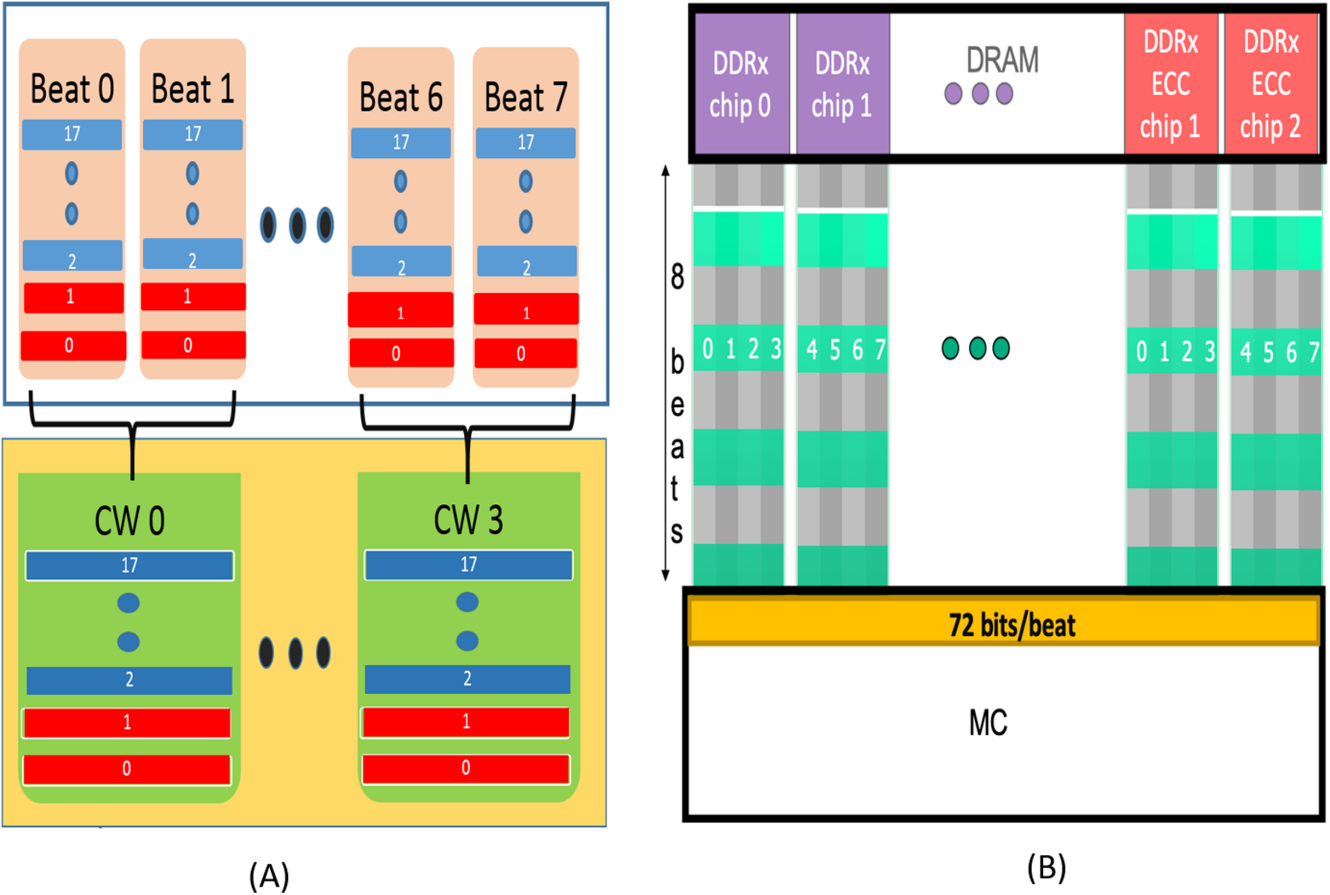
(A) AMD’s SSC Design. Each Codeword is formed by interleaving 2 successive beats. Each codeword has two check symbols (Red) and 16 data symbols (blue). (B) QPC-Bamboo. Each symbol is composed of 8 bits transferred by a single pin. QPC-Bamboo uses one codeword composed of 72 such symbols for the entire cache-line. Each beat comprises of 72 bits (64 from data chips and 8 from ECC/redundant chips), one from each symbol.

During a WRITE operation, the MC divides the 64-byte cache line into four data blocks of size 128 bits each (16-data symbols). Each data block is encoded with RS-SSC to generate 16 check bits (2 check symbols). Each codeword therefore has 16 data symbols and 2 check symbols with a redundancy of 12.5%. As shown in Fig. 2A, each symbol of the codeword is split into two blocks. For example, symbol 17 in CW 0 is split into two 4-bit blocks assigned to 17th block in Beat 0 and Beat 1 respectively in the figure. The 17th block of “Beat 0” has all odd bits of the symbol and the 17 block of “Beat 1” has all the even bits of symbol 17. In this way, each beat is composed of 16 data blocks (shown in blue) meant to be stored in 16 devices, and 2 blocks (shown in red) from check symbols of the encoded codeword, meant to stored in the two redundant devices. The remaining 3 datablocks are also encoded and transferred via 6 beats. So, each cache-line makes use of four codewords and 8 beats.

During READ operation, bits from two successive beats are interleaved at the MC to form one codeword. By employing RS-SSC decoder, the MC provides “Chipkill” functionality as each symbol now contains bits from same ×4 device in the rank. In this way, all the four codewords are decoded at the MC. If any device fails or bits stored in the device are corrupted, at-least one symbol corresponding to the device gets corrupted and can be recovered by using SSC-RS decoding.

This design is used as our baseline for comparison. When there are more than one symbol with errors per codeword (mostly due to multiple chip failures), AMD uses history based hardware-software approach to cover these scenarios.

#### Three check symbol based SSCDSD (Single symbol correct, double symbol detect) CHIPKILL

Single Symbol Correct, Double Symbol Detect can also be implemented with three check symbols. For example, Lin & Costello (1984) describes an extended Reed Solomon code (*n* + 3,*n*,8) with three check symbols. For current ×4 DDR4 systems, it is possible to employ a RS (19,16,8) code with 19 devices devices per rank (64 data + 12 redundant bits/beat) and 76 bit data bus. Similar to AMD’s SSC CHIKPILL, this scheme can also combine two successive beats to form one codeword. Each cache request (64 bytes) can make use of four codewords to provide SSCDSD capability. We compare the tradeoffs between this scheme and our solution in the “Evaluation”.

### DDR4 bus reliability mechanisms

#### WRITECRC

In DDR4, WRITECRC is designed to detect transmission errors in data during WRITE operation. In this design, the memory controller generates an 8-bit CRC checksum for the entire write data burst (8 beats) to each chip/data-lane (JEDEC, 2020a), of the rank. These 8 bits are sent over two additional beats after the data is sent to the individual chips. Each DRAM chip includes logic to re-compute the CRC checksum and compare it with checksum received from the controller. Such a design allows the chips to detect errors before storing them and provides an option to retry the transmission of the data. However, transmission errors during READs (not covered by WRITECRC) may also lead to SDCs with the baseline scheme.

#### CA (Command/Address) parity

Command/Address parity uses an additional pin (or bus-line) to transfer even parity of the CMD/ADD signals to every DRAM chip. It cannot detect an even number of bit-errors on the CMD/ADD signals.

#### Data bus inversion

Data Bus Inversion is designed to protect against Simultaneously Switching Noise (SSO) (Mentor Graphics, 2015), during data transmission for ×8, ×16 DDR4 chips. With 8 Data bits/pins and an additional 9th pin per each data-lane, DBI (Data Bus Inversion) ensures that at least 5 out of 9 pins are “1”s. This avoids the situation where all bits switch from 0 to 1 or vice-versa to improve the signal integrity of data bus.

#### Gear down mode

Gear-down mode allows the MC to lower transmission rate of command/address and control signals to trade-off latency and command bandwidth for signal quality while maintaining high data rates.

### Memguard Chen & Zhang (2014)

Memguard is a reliability scheme designed to detect multi-bit errors in DRAMs without using redundant storage. It makes use of two registers (READHASH, WRITEHASH) and custom logic at the memory controller (MC). Whenever there is a memory transaction between the last level cache and the DRAM, the logic at MC computes a hash value for this transaction and READHASH/WRITEHASH registers are updated. This scheme does not store the hash values in the memory and uses an incremental multi-set hashing technique (Clarke et al., 2003). By periodically synchronizing the two hash registers at the MC, Memguard detects any error that occurred during that period and relies on OS-checkpointing for error recovery.

Although this scheme can detect multi-bit (or multi-symbol) errors, on its own it is not suitable for HPC/datacenters due to the high recovery time associated with checkpointing and synchronization. Also, Memguard is effective only against soft errors. Although, we do use a hash function in our scheme, our purpose is completely different. We do not use incremental multi-set hashing technique and we store hash along with data and ECC bits in the DRAM for redundancy. Thus, we employ ECC and hash to provide error detection and correction for each cache-line read and write, and do not require any synchronization over time. This ensures faster recovery, effectiveness against both permanent and soft errors, and is therefore suitable for HPC data centers and servers.

### QPC bamboo ECC (Kim, Sullivan & Erez, 2015)

SECDED and CHIPKILL designs described earlier orient the symbols parallel to the beats. This will reduce the latency overhead associated with decoding as the memory controller can perform the decoding on the received codeword while waiting for the remaining codewords. This work argues for aligning the ECC symbols orthogonal to the beats to provide improved reliability at the cost of increased latency.

QPC Bamboo provides CHIPKILL capability (four/quadruple pin correction capability) with 12.5% redundancy for ×4 based memory systems. As shown in Fig. 2B, this scheme makes use of 8 bits of the 64-byte cacheline transferred through a pin of an ×4 device as one symbol. With two additional devices per rank (12.5% overhead), there will be a total of 72 symbols per cache-line. They use one codeword (size—72 symbols) of RS-Single Symbol Correction Code for the entire cache-line. Of these, 64 (16 × 4) are data symbols from 16 devices and 8 (2 × 4) are check symbols from two additional devices. This RS-SSC code can correct up-to 4 (8/2) erroneous symbols. Based on their error model, they show that QPC-Bamboo provides stronger correction and safer detection capabilities than AMD’s CHIPKILL (SSC (Single Symbol Correction) CHIPKILL). Hence, we also evaluate QPC Bamboo in our comparative studies.

Our goal in this article is to consider more realistic error model based on the nature of faults and develop an appropriate scheme to protect against them.

### AIECC-all inclusive ECC (Kim et al., 2016)

AIECC is a suite of mechanisms designed to protect against clock, control, command, and address (CCCA) faults without additional redundant storage or new signals.

Our work is orthogonal to AIECC scheme. We focus on improving detection capability against device, data and address errors while AIECC focuses on CCCA errors with limited protection against address errors. The reliability of future memory systems can be improved by incorporating our solution along with AIECC.

## Preliminary Motivational Experiments

In the presence of an error, a generic reliability scheme reports it as either a Correctable Error (CE) or a Detectable but Uncorrectable Error (DUE). When an error is outside of the coverage of the scheme, it can result in a Detectable but Miss-corrected Error (DME) or an Undetectable Error (UE). DMEs and UEs are collectively called as Silent Data Corruptions (SDCs) as they do not raise an alarm.

The baseline scheme uses RS (18, 16, 8) systematic SSC code. A RS (n, k, m) codeword has k data symbols and n-k check symbols with m bits per symbol. The minimum hamming distance between any two codewords is *n* − *k* + 1 (3 in this case). It can correct : ⌊(*n* − *k*)/2⌋ (1 for baseline scheme) symbol errors. When there is an error across multiple symbols of a codeword, the RS decoder can either identify it to be uncorrectable error (DUE) or “miss-correct” it to another codeword thinking it to be a single symbol error of another codeword (DME) or fail to detect presence of the error (UE). As a result, multiple symbol errors can result in Silent Data Corruptions (collective term for DMEs and UEs) in the baseline scheme. We experimented to assess the level of Silent Data corruptions in the baseline scheme in the presence of multi-symbol errors.

We developed an in-house simulator to perform our experiments. We used open source software (Minsky, 2013; Rockliff, 1991; Eruchalu, 2014) to develop Galios (symbol-based) arithmetic, RS encoder and decoder. We use generator polynomial—*G*(*x*) = (*x* − *a*^1^)(*x* − *a*^2^)…(*x* − *a*^*N*^) (where *N*—number of ECC symbols/CW) to construct RS code. Our decoder uses Berlekamp Massey algorithm for correcting/detecting errors.

For each iteration of the experiment, we generated random 16 byte dataword and used RS encoder to generate 18-symbol codeword. We stored this codeword in an array. With the help of 18-symbol error mask, we inserted errors into the stored codeword. We then, decoded the stored codeword (with errors) using the RS-decoder. The decoder flagged whether each codeword had “No Errors” or “Detectable but Uncorrectable Errors” or “Correctable Errors”. If the decoder detected a correctable error in a codeword, it corrected the corresponding stored-codeword. Next, we retrieved the stored data word processed by RS-decoder and compared it with the original data word to identify silent data corruptions.

We executed three experiments—introducing random 2, 3 and 4 symbol errors per codeword. Each of these experiments was run for ten iterations with 1 billion random datawords in each iteration. Table 1 lists the mean % across 10 iterations for the number of miscorrections (DMEs), detected but uncorrectable errors (DUEs) and undetected errors (UEs) with Berlekamp Massey algorithm based RS decoder. The standard deviation for each of the experiments (except for UEs with random 2 symbol errors) was up to 13,000. In the table, we provide the mean of UEs (in parenthesis) along with mean % of UEs.

**Table 1:** Results of Random multi-symbol data errors for RS (18,16,8).

Experiments	Miscorrected (DMEs) (%)	Detected but Uncorrected (DUEs) (%)	Undetected (UEs) (%)
2 Symbol Errors/CW	6.3	93.7	0% (0)
3 Symbol Errors/CW	6.9	93.1	−> 0% (∼10,000)
4 Symbol Errors/CW	7.0	93.0	−> 0% (∼10,000)

Due to simplicity of hardware design, most of the hardware implementations use either algorithm based on Euclidean approach or Berlekamp Massey, to implement RS decoder. We therefore, performed these simulations with Euclidean based RS decoder as well. The results were similar to what we observed in Table 1 with the BM algorithm. The mean percentages of DMEs and DUEs for Euclidean decoder for all the three experiments were less than 0.5 percent away from what we observed with Berlekamp Massey (Table 1). The mean percentage of UEs for all the experiments were identical to the results shown in Table 1.

### Theoretical analysis

**Figure 3:**
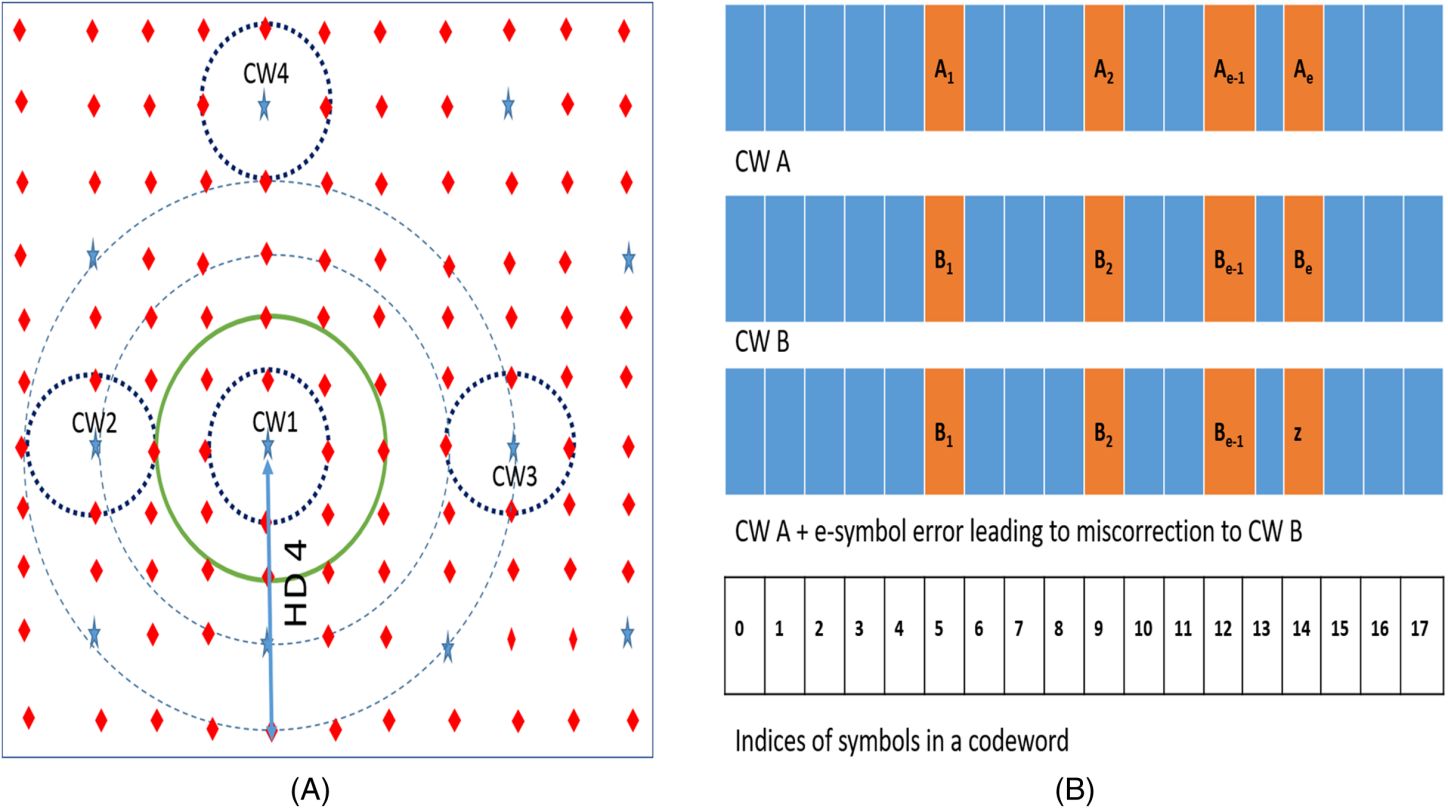
(A) N tuple space representation of Reed Solomon SSC code. Stars are codewords which are at least 3 Hamming Distance apart. (B) CWs A and B are two codewords at HD = *e*. In the depiction, *e* errors in CW A may place it at HD = 1 from CW B.

We can explain the results of our experiments described in the “Preliminary Motivational Experiments” with the help of an analytical method (Sofair, 2000). Figure 3A depicts the codespace of the baseline RS (18, 16, 8) code. In the figure, stars represent valid codewords and diamonds represent non-codewords. Due to errors, a particular codeword (say CW1) gets corrupted and may be detected by RS decoder as a non-codeword (diamond) or as other codeword in the space (Kim, Sullivan & Erez, 2015). The dotted hypersphere which is HD = 1 away from codeword represents the correction range of the SSC. All the words on this sphere will be corrected to the codeword on the center of the sphere (in this case CW1). Words on HD = 2 hypersphere (solid line in green) are either detected as errors or miscorrected to the adjacent codeword. Words on the dashed sphere (HD = 3) are either correctly detected as errors or undetected (falsely detected as adjacent codeword) or miscorrected as another codeword.

For a generic RS (*n*, *k*, *m*) code, the total n-tuple space available is 2^*n*^ * ^*m*^. Out of this space, the number of codewords are 2^*k*^ * ^*m*^. Assuming that the space is uniformly distributed among the codewords, we can say that the space around (or owned by) each codeword is 2^*n*^ * ^*m*^/2^*k*^ * ^*m*^.

If we introduce “*e*” symbol errors from a given codeword (say CW1), all such words lie on a hypersphere at HD = *e* from the codeword. If “*e*” is greater than minimum HD of “*n* − *k* + 1”, this sphere may also contain other codewords. For example, as shown in Fig. 3A, the RS code has two codewords (CW1 and CW2) which are HD = 3 apart. If we introduce 4 symbol errors from CW1, the hypersphere centered on CW1 with radius 4 also contains CW2. On an average, the number of such codewords *C*_*e*_ on or inside a hypersphere HD = *e* away is approximately given by dividing the total number of words inside the sphere by number of words "owned" by each codeword is as follows.

(1) $${C_e} = \displaystyle{{\sum\nolimits_{{\rm{\alpha}} = 1}^e {n_{{C_{\rm{\alpha}} }\left( {{2^m} - 1} \right)}}{\rm{\alpha}} } \over {{{\left( {{2^m}} \right)}^{n - k}}}} - 1$$

The RS decoder “mis-corrects” such an “e” (where $e > (n - k + 1)$) symbol error from a given codeword when the e-symbol error also: (1) falls on HD = 1 sphere of another CW which is HD = *e* away OR (2) falls on HD = 1 sphere of another CW which is HD = *e*, − 1 away OR (3) falls on HD = 1 sphere of another CW which is HD = *e* + 1 away. For example, Fig. 3A shows a hypersphere at HD = 4 away from CW1. This sphere represents all the 4-symbol errors from CW1. Few words on this sphere get mis-corrected to CW3, which is at HD = 4 away from CW1. Due to presence of CW2 at HD = 3 away from CW1, few other words on sphere HD = 4 also fall on HD = 1 sphere of CW2 and therefore get mis-corrected. Similarly, few other words on this HD = 4 sphere also fall on HD = 1 sphere of CW4 which is at HD = 5 away from CW1.

Using Eq. (1), the number of CWs at HD = *e* from a given codeword is given by ${C_e} - {C_{e - 1}}$ which is equal to

(2) $$\displaystyle{{{n_{{C_e}\left( {{2^m} - 1} \right)}}e} \over {{{\left( {{2^m}} \right)}^{n - k}}}}$$

Now, due to the presence of one CW at HD = e from a given CW A, more than one “*e*” symbol errors are miscorrected. Figure 3B shows two codewords CW A and CW B which are HD = *e* away. There will be exactly ${e_{{C_{e - 1}} \cdot \left( {{2^m} - 2} \right)}}$ number of “*e*” symbol errors from CW A which are HD = 1 away from CW B and hence will be miscorrected to CW B. Combining this with Eq. (2) we get the expression for total number of “*e*” symbol errors from a given codeword CW A that will be miscorrected due to presence of codewords at HD = e from CW A as follows.

(3) $${m_e} = \displaystyle{{{n_{{C_e}}} \cdot {{\left( {{2^m} - 1} \right)}^e} \cdot {e_{{C_{e - 1}}}} \cdot \left( {{2^m} - 2} \right)} \over {{{\left( {{2^m}} \right)}^{n - k}}}}$$

Similarly, we can calculate number of “*e*” symbol errors that will be miscorrected due to presence of codewords at HD = *e* − 1, HD = *e* + 1 given by Eqs. (4) and (5), respectively.

(4) $${m_{e - 1}} = \displaystyle{{{n_{{C_{e - 1}}}} \cdot {{\left( {{2^m} - 1} \right)}^{e - 1}} \cdot {{\left( {n - e + 1} \right)}_{{C_1}}} \cdot \left( {{2^m} - 2} \right)} \over {{{\left( {{2^m}} \right)}^{n - k}}}}$$

(5) $${m_{e + 1}} = \displaystyle{{{n_{{C_{e + 1}}}} \cdot {{\left( {{2^m} - 1} \right)}^{e + 1}} \cdot {{\left( {e + 1} \right)}_{{C_e}}}} \over {{{\left( {{2^m}} \right)}^{n - k}}}}$$

The total number of “*e*” symbol errors from a CW is given by ${n_{{C_e} \cdot \left( {{2^m} - 1} \right)}}e$. Therefore, the fraction of miscorrections in the set of “*e*” symbol errors from a CW is given by m_total_.

(6) $${m_{\rm total}} = \displaystyle{1 \over {{n_{{C_e} \cdot \left( {{2^m} - 1} \right)}}e}} \cdot \left( {{m_e} + {m_{e - 1}} + {m_{e + 1}}} \right)$$

Using Eq. (6), we calculate the fraction of miscorrections. For the first experiment (Random 2 Symbol errors) as RS (Tang et al., 2016; Kotra et al., 2017; Siddiqua et al., 2013) code has a minimum HD of 3, there are no codewords at, as the code-space is sparsely populated, 93.7% of random errors on HD = 2 sphere do not fall on HD = 1 spheres of other codewords. Also, as expected, we do not observe any undetected errors in this experiment as there are no codewords at HD = 2. Similarly, we calculate the fraction of miscorrections for the second and third experiments and find that these also corroborate with the experimental results in Table 1. The total information space available for single symbol correcting RS (18,16,8) is 2^18×8^(2^*k*×*m*^). Out of this, 2^16×8^(2^*k*×*m*^) are to be used as codewords. As the fraction of codewords over the total space is only 2−16(2^16×8^/2^18×8^), as the code-space is sparsely populated, 93.7% of random errors on HD = 2 sphere do not fall on HD = 1 spheres of other codewords. Also, as expected, we do not observe any undetected errors in this experiment as there are no codewords at HD = 2. Similarly, we calculate the fraction of miscorrections for the second and third experiments and find that these also corroborate with the experimental results in Table 1.

As we are able to corroborate the experiment results with our analytical model, we have confidence that our experimental framework is able to accurately simulate Reed Solomon decoder and random error injection. Also, these results further motivated us to develop a solution to tackle SDCs in current and future DRAM subsystems.

## Error Model

To represent the possible fault modes that may occur in current/future DRAM systems, we first describe our error model. This model covers various type of faults that arise in DRAM devices, data-bus and address-bus.

Faults in DRAM subsystems are caused due to a variety of sources such as cosmic rays (Baumann, 2005), circuit failure, signal integrity etc. These faults can be broadly categorized as transient or permanent. Transient phenomena corrupt memory locations temporarily, once rewritten these locations are free from errors. Permanent faults cause the memory locations to consistently return erroneous values.

Field (Meza et al., 2015; Sridharan et al., 2015; Schroeder, Pinheiro & Weber, 2009; Hwang, Stefanovici & Schroeder, 2012) help us in understanding the trends of errors in DRAM subsystem up to a certain extent. We use this information along with nature of faults in DRAM subsystem to develop our error model (Table 2). Here, we describe the sources of these faults and the corresponding errors perceived per cache-line due to a particular fault type. Single bit errors are due to failures in DRAM cells or due to faulty column. Due to failure in a sub-array or one DQ pin (one bus line in a bus-lane), bits transferred over a single DQ pin are corrupted. Failures in circuitry inside chips such as sense amplifiers, address decoders etc. cause particular rows/columns/banks/chips to malfunction. For example, if a local row buffer (sense-amplifier) in a bank of a chip is stuck at 1, then all the bits fetched from the chip of particular READ request are read as “1”. Therefore, each word (bits provided by a chip in one beat) fetched from this chip will have all 1’s for this particular READ.

**Table 2:** Different faults and the corresponding Error Model.

Fault mode	Source	Error pattern per cacheline
1 bit/Column	Particle strike/cell or column failure	1 bit error
1 pin/Sub-array fault	Fault in 1DQ of a buslane, or subarray	1 pin stuck at 0 or 1 for all beats
Row/Chip/Bank fault	Failure of sub-array row driver/address decoding circuit	1 word related to faulty chip stuck in all beats
Bus fault	Fault in 1 bus lane	errors in random beats of a bus
Correlated Bus fault	External noise or Cross -coupling	Consecutive Bus-lane faults
1 bit/pin + other faults	Both 1 bit/pin + pin/row/chip/bus	faults lead to 2 symbol errors
Chip + Chip	2 faulty chips OR byzantine address faults	2 specific words in all beats stuck at 1 or 0 or random pattern
3 fault mode	byzantine address faults OR combine 3 of above faults	Random errors in 3 words/beat
multi-symbol fault mode	byzantine address faults in >3 devices/rank	Random errors in >3 words/beat
Address fault	Fault in MC-DIMM address bus	Memory Data corruptions during WRITE fetch data from unintended address during READ

Bus faults are another source of errors. According to 1st order analysis, bus lines act as a low pass filter. Since digital signals are composed of numerous frequencies, distinct components of these signals experience attenuation to a different degree giving rise to signal degradation. Reflection is another first order effect which results in signal degradation.

Table 3 describes other sources of transmission faults (Jacob, Ng & Wang, 2007) and their impact on signal integrity of the data bus. As most of the errors associated with bus faults are data-dependent or random, we expect random errors in different beats of a faulty data-bus. To simulate this behavior for a single data-bus fault, we use a random number to identify the erroneous beat positions among eight beats. We then inject random errors in these positions. We also consider correlated data-bus fault due to presence of external noise or coupling between two bus lanes. In this fault-mode, we expect two neighboring data-bus lanes to be faulty. Similar to single bus fault, we first identify erroneous beat positions and inject random errors for these two neighboring data-bus lanes.

**Table 3:** Summary of Data Transmission faults.

Transmission Fault	Description/Cause	Impact on Signal Integrity
Dielectric Loss	Signals attenuate as a function of trace length and frequency	All data bits are affected, results in signal attenuation
Skin effect	Resistance of conductor varies non-uniformly with frequency	All data bits are affected, results in signal attenuation
Electromagnetic interference	Electromagnetic/capacitive coupling of closely packed lines	few bus lines/lanes are affected at one point of time
Skew	Path length variations result in timing variations	Random
Jitter	Fluctuations in voltage, temperature and crosstalk between successive cycles of a given signal impact the propagation time of the signal	Difficult to model/characterize
Inter symbol interference	Past signals on a line have residual effects on subsequent signals of the same line	Random, Data dependent
Simultaneously switching output	When many signals in a bus-lane switch, they induce coupling on other signals	Data dependent.

We also combine single bit/pin faults (as they occur with higher frequency (Sridharan & Liberty, 2012) with other fault types to model 2-symbol/chip errors per codeword. Note that 1bit/pin fault + other faults lead to either 1 or 2 symbol errors per codeword. Based on our preliminary experiments, we are interested in scenarios which lead to 2-symbol errors per codeword (1 symbol errors are anyways handled by existing chipkill mechanisms), therefore, in our experiments for 1-bit/pin + other faults, we insert errors in 2 different symbols for the codewords. This results in 2 symbol errors/CW.

**Figure 4:**
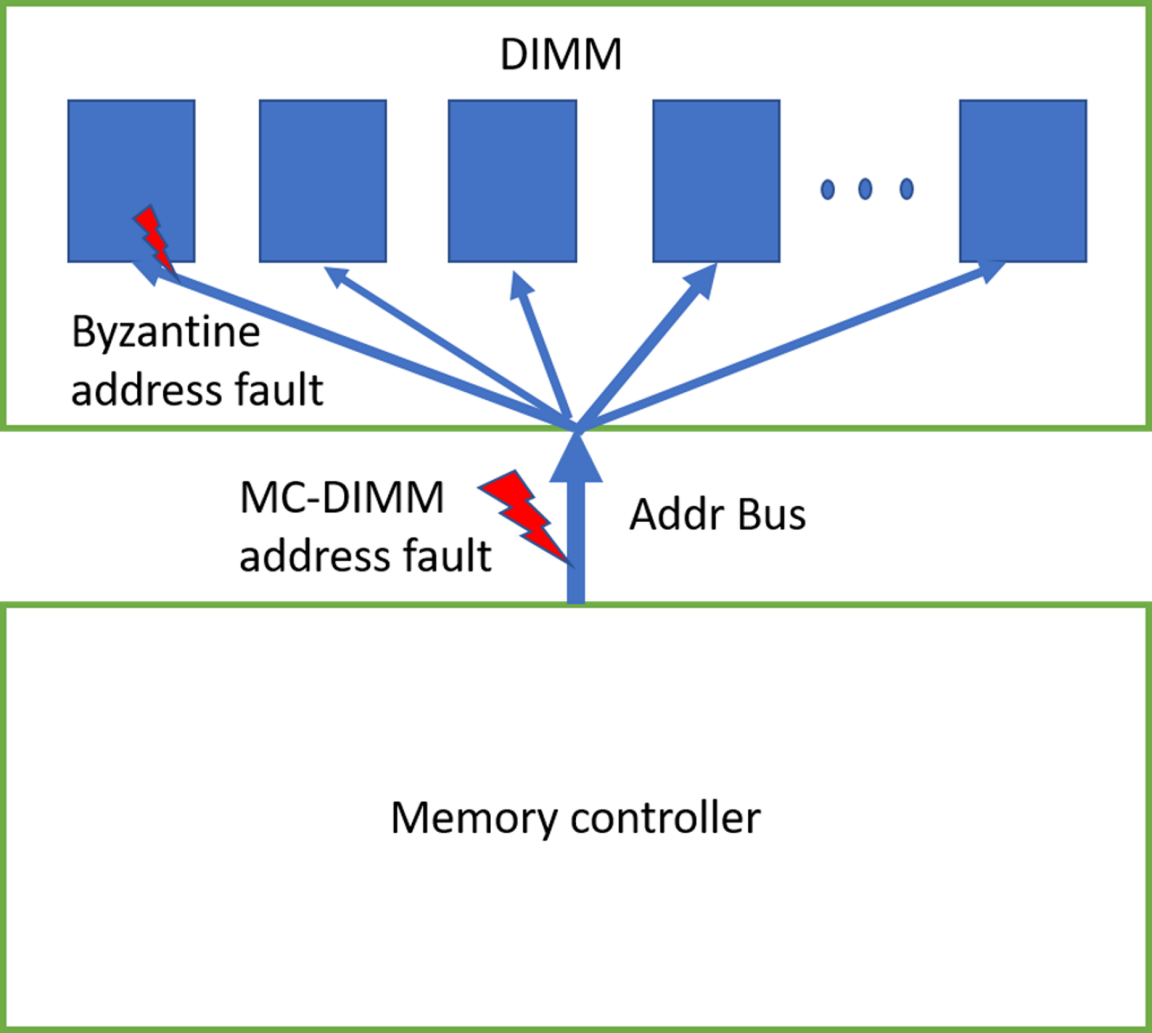
DDRx Address bus. We can have MC-DIMM address bus faults and byzantine faults. Bus topology inside the DIMM/rank is not shown in the figure.

Address-bus is also prone to faults. These faults can be primarily categorized into two types. In one fault mode, all the DRAM devices of the rank receive the same (erroneous) address. This happens when there is a fault in the address bus in-between the MC and DIMM (shown in Fig. 4). We also consider another type of byzantine fault, where few of the devices in the rank receive erroneous addresses. As the address bus inside the DIMM drives all the devices of the rank, it is prone to byzantine faults—where due to unstable bus lines, different devices sense the address sent on the address-bus differently. Note that DIMM manufacturers use different type of bus topologies such as fly-by (JEDEC, 2020a), tree, hybrid tree, but we do not show them in the figure.

When devices in the rank receive erroneous address during WRITE operation, there is memory data corruption (Kim et al., 2016). When only one of the devices receives erroneous address (due to byzantine fault) during WRITE, one symbol/CW is corrupted and therefore ECC deployed by baseline can recover it. If >1 device receive erroneous address, it will lead to >1 symbol error/CW, hence it could result in detected but uncorrectable error (DUE) or detected but miscorrected error (DME) or undetected error (UE).

**Figure 5:**
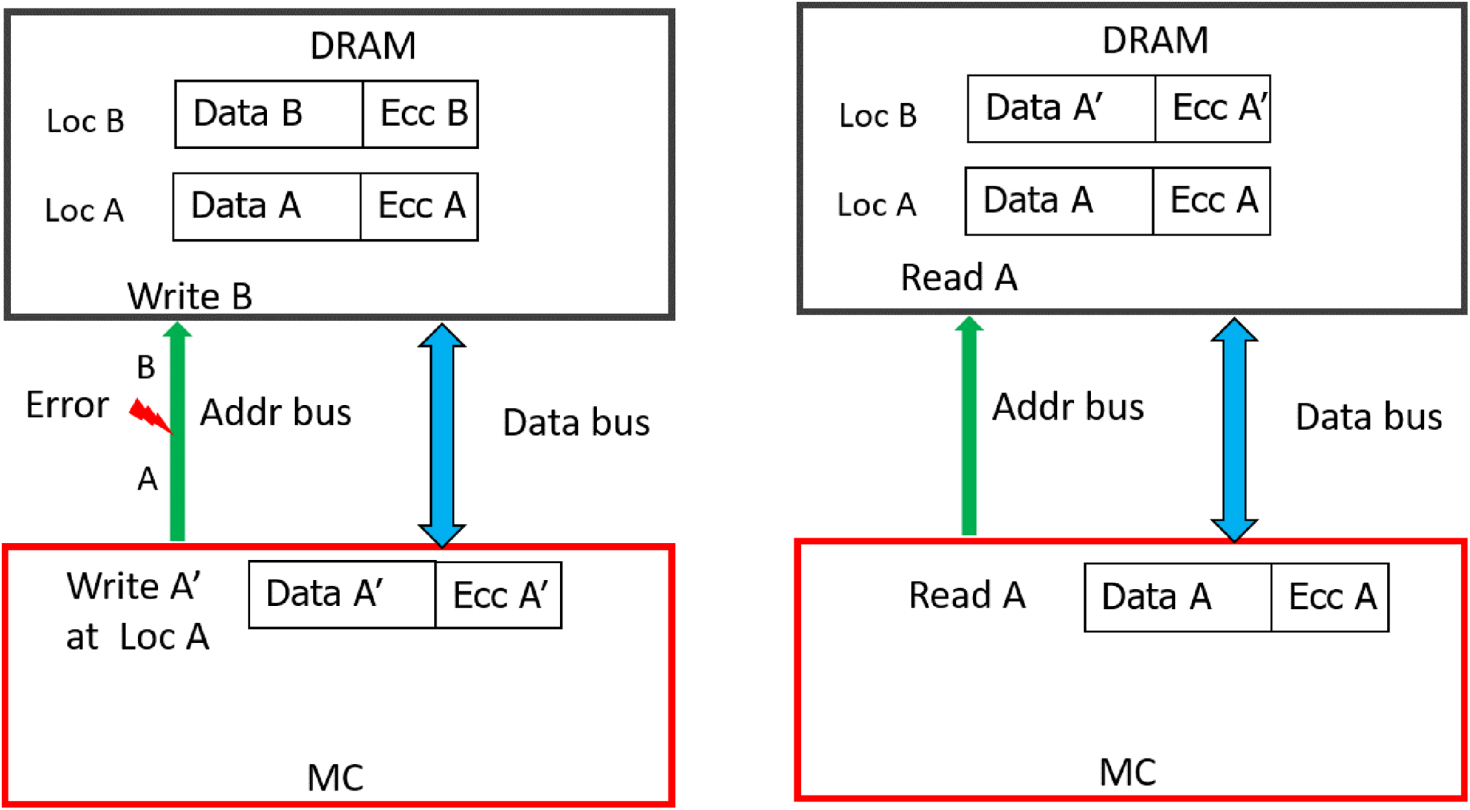
Memory Data corruption (for all the DRAM devices) due to MC-DIMM address fault during WRITE operation with the baseline.

As shown in Fig. 5, due to address fault in-between the MC and DIMM, during WRITE operation (WRITE address A), all the DRAMs of rank register this as a WRITE operation to location B. As a result, the contents of location B are corrupted (with A′) and location A has stale data (A instead of A′). Therefore, untill these locations are written back again without errors, READs to both locations A and B will lead to Silent Data Corruptions (Undetectable Errors). In the figure, a subsequent READ request (without error) to location A, yields the stale data (A, not A′). This is because, the baseline CHIPKILL scheme does not keep track of address associated with the data, it will decode the codewords and inadvertently pass the data from address location to entity (I/O or processor) which initiated this READ request.

To prevent such memory data corruptions, JEDEC has introduced CAP-Command Address Parity (JEDEC, 2020a), in DDR4, so that errors are detected before writing into the DRAM. Another recent work, AIEC (Kim et al., 2016) proposed a stronger protection mechanism called eWRITECRC to address this concern.

**Figure 6:**
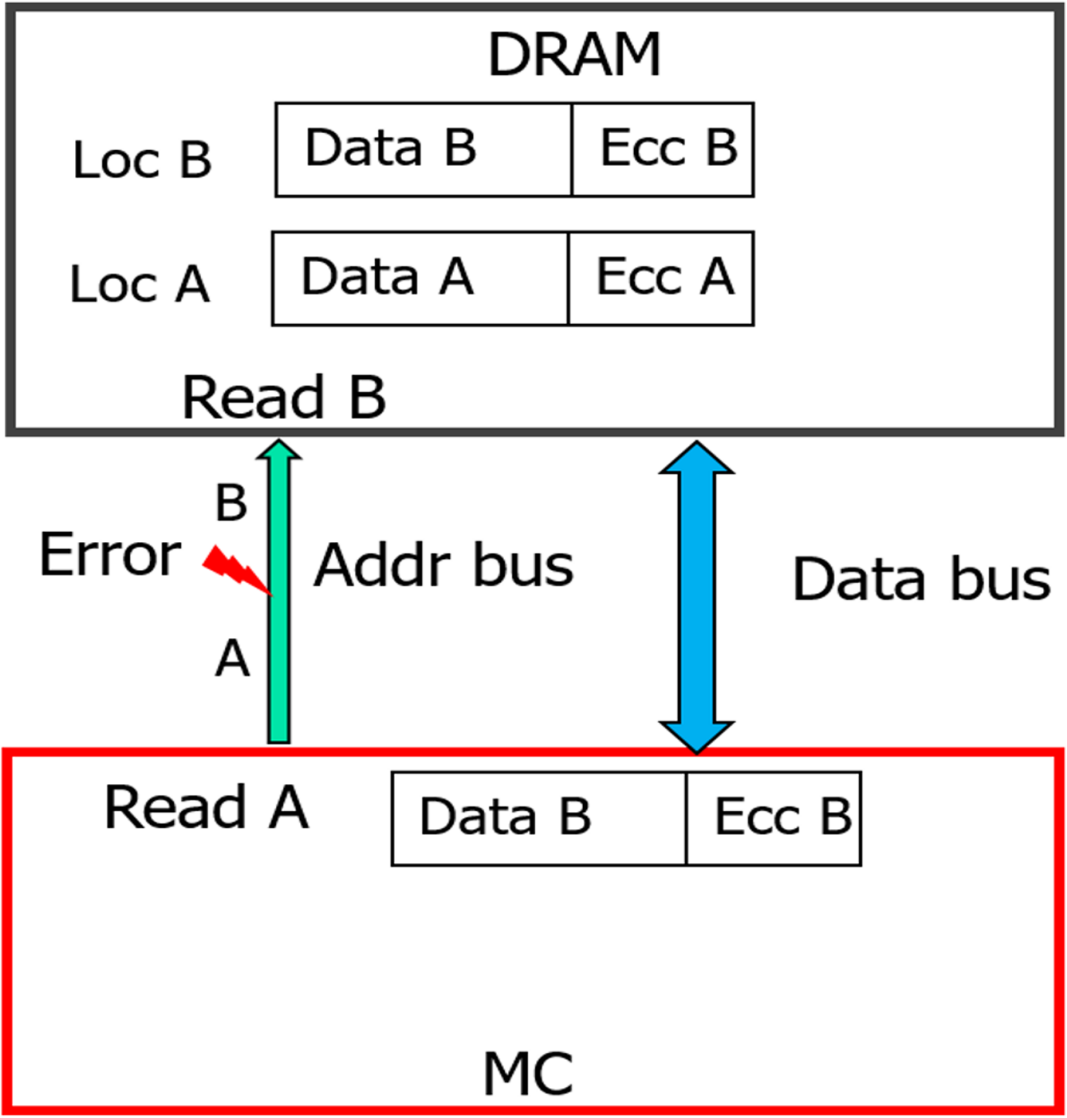
Silent Data corruption (for all the DRAM devices) during READ operation with baseline.

With weaker CAP (can detect only odd-bit errors), errors in address bus during READs can result in Silent Data Corruption. Similar to WRITE operation, if >1 device receives erroneous address, it could result in DUE or DME or UE. But if only one of the devices receives erroneous address (byzantine fault), the ECC deployed by baseline can recover it as only one symbol/CW gets corrupted. As shown in Fig. 6, due to fault in MC-DIMM address-bus during READ operation (READ address A), all the DRAMs of rank register this as a READ operation to location B. Therefore, the MC receives codewords from an incorrect address (address B). To provide stronger protection for up-to 32 address bits, eDECC was introduced in Kim et al. (2016).

As address faults in the MC-DIMM interface leads to SDCs, we include them in our error model. Byzantine address faults lead to multiple (2, 3 or more) symbol errors/CW. There is also possibility for other faults to occur simultaneously across three different chips/data-bus lanes. To consider such multi-symbol error scenarios, we include 3-fault mode and multi-symbol fault mode in our error model.

## SSCMSD—A Novel Architectural Solution for Multi-bit/Multiple Symbol Errors

We first carried out a set of experiments detailed in our error model to study the behavior of the baseline (SSC-RS (Tang et al., 2016; Kotra et al., 2017; Siddiqua et al., 2013)) scheme. The results are shown in the column labeled Baseline RS(18,16,8) in Table 5 in “Evaluation”. As described earlier, as the code-space is sparsely populated, this scheme can detect many multi-symbol errors as well. However, as shown in this table, the baseline is still prone to SDCs with multiple device, data-bus and address-bus faults. Inspired by this observation, we chose to further decrease this SDC rate by improving the ECC scheme at the memory controller with minimal increase in redundancy, that is, 1 more redundant chip and corresponding bus lane.

As described in “3 Check Symbol Based SSCDSD (Single Symbol Correct, Double Symbol Detect) CHIPKILL”, one can use another redundant chip to have a total of three check symbols per codeword (baseline uses two check symbols per codeword) to provide SSCDSD capability. The column labeled RS-SSCDSD shows the performance of this extended scheme with our error model. As expected, all the double symbol errors are detected by this scheme, but it is still prone to SDCs with greater than 2 symbol errors and address protection can be improved.

An interesting point to note from these results is that the SDC rate is dependent on the type of error pattern a fault generates rather than on the number of bits/symbols being corrupted. For example, 1 bit + Chip fault corrupts 9 bits per CW and has 6% SDC rate while 1-bit fault + 1-pin fault corrupts 3 bits of a particular CW and has a SDC rate of 7.6% for the baseline scheme. Although we do not show the breakdown of SDCs into UEs and DMEs for the baseline in Table 5, our evaluation shows that for all the experiments of baseline and Extended-baseline schemes, SDCs occur mostly (99%) due to miss-corrections (DMEs) from the SSC-RS decoder. Therefore, the stored information is subjected to errors from faults and due to errors induced by the decoder. These observations warrant inclusion of additional mechanisms to be developed and included in the memory systems. Our solution is to use a hash function, as the hash value allows us to identify such arbitrary corruption. We use a non-cryptographic hash function to compute a signature of the data, address. We use this signature to detect multi-bit errors and address errors with high probability. By combining hash and CHIPKILL, we develop our new error handling scheme, called Single Symbol Correct, Multiple Symbol Detect (SSCMSD) CHIPKILL.

**Table 4:** Possible scenarios after Hash computation and Syndrome calculation.

Hash check	Syndrome calculation	Decision
H1 = H′	S_*i*_ = 0 for *i* = 1 to 4	Declare Error Free
H1 = H′	at least one of S_*i*_ != 0	Error, Try to correct it with SSC-RS and check back with hash
H1 != H′	S_*i*_ = 0 for *i* = 1 to 4	Declare Error
H1 != H′	at least one of S_*i*_ ! = 0	Error, Try to correct it with SSC-RS and check back with hash

**Table 5:** Comparison of SSC-RS, Bamboo-ECC and SSCMSD.

Comparison	Baseline RS (18,16,8)	Bamboo-ECC RS (72,64,8)	RS-SSCDSD (19,16,8)	Extended Bamboo RS (76,64,8)		SSCMSD RS (19,17,8) & 32-bit hash	Stats
				v1	v2		
Storage Overhead (%)	12.5	12.5	18.75	18.75		18.75	
ECC Symbols/CW	2	8	3	12		2 + 1	
CWs/Cacheline	4	1	4	1		4	
Up to 1-Chip/Bus Fault	100	100	100	100	100	100	CF
Correlated Bus fault	2.0	11.4	0	12.3	0	0	SDC
	98.0	88.6	100	87.7	100	100	CF
1 bit fault + 1 bus fault	4.0	11.1	0	0	0	0	SDC
	96.0	88.9	100	100	100	100	CF
1 bit fault + (row/bank/chip)	6.0	11.0	0	0	0	0	SDC
	94.0	89.0	100	100	100	100	CF
1 bit fault + 1 pin fault	7.6	0	0	0	0	0	SDC
	92.4	100	100	100	100	100	CF
1 pin fault + 1 pin fault	3.5	0	0	0	0	0	SDC
	96.5	100	100	100	100	100	CF
Chip fault + Chip fault	<0.1	11.0	0	12.0	0	0	SDC
	>99.9	89.0	100	88.0	100	100	CF
3 fault mode	<0.1	11.0	<0.1	12.0	~10	0	SDC
	>99.9	89.0	>99.9	88.0	~90	100	CF
multi-symbol (>3) fault mode	<0.1	11.0	<0.1	12.0	~10	0	SDC
	>99.9	89.0	>99.9	88.0	~90	100	CF
MC-DIMM Address fault Detection during							
WRITE	Odd bit	Odd bit	Odd bit	Odd bit		Odd bit	
READ	Odd bit	Odd bit	Odd bit	Odd bit		Random	
Latency overhead (READ)	+1	+4	+1	+4		+1	

We describe the design of our scheme below. For the sake of clarity, we discuss the design space of using hash of only the data and then include the address.

**Figure 7:**
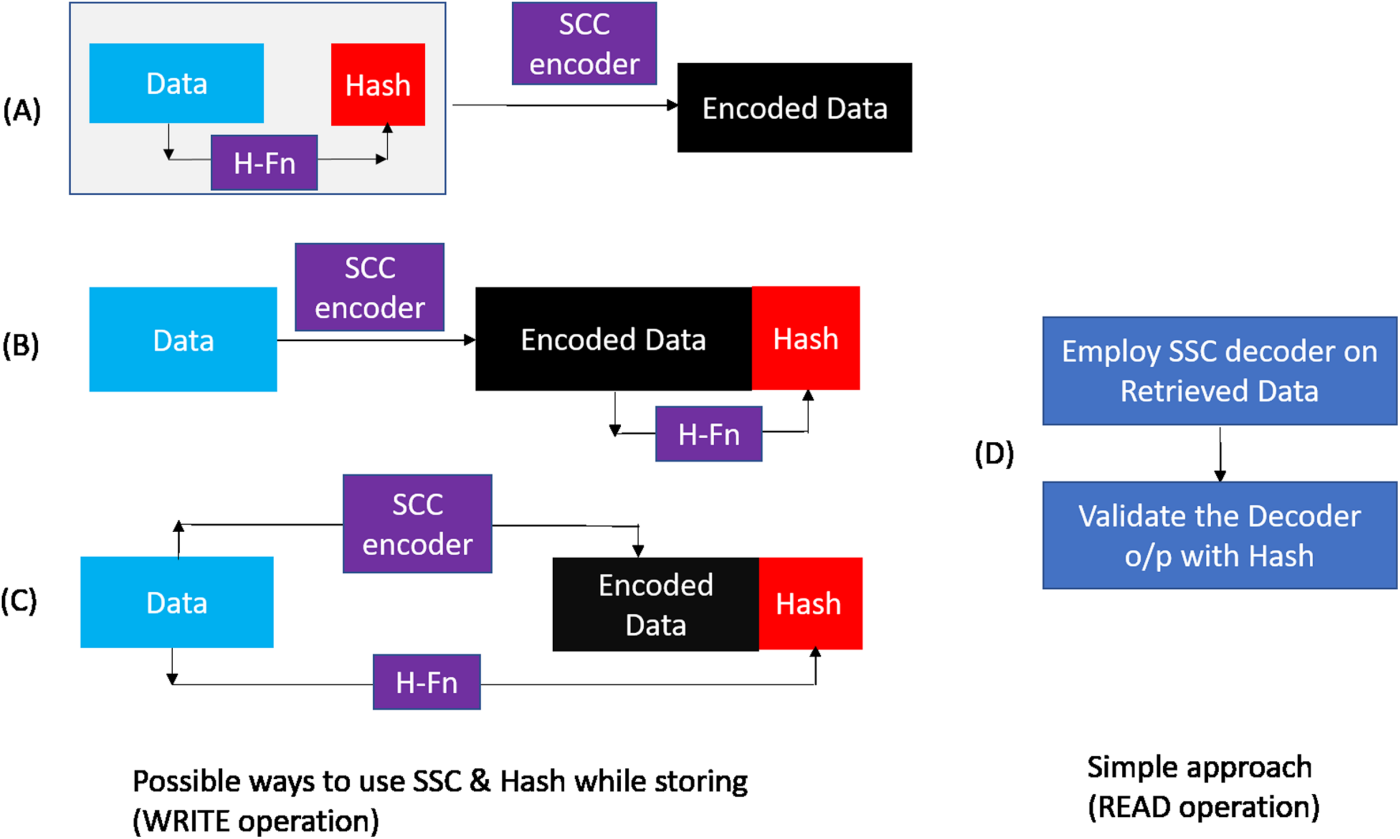
Possible hash and SSC combinations. Different means to combine SSC & Hash during WRITE operation: (A) Compute the hash of data and then use SSC encoder to encode data and hash. (B) encode the data and then compute the hash of encoded data. (C) encode the data and compute the hash of data in parallel During READ, (D) shows the Simple approach to employ the SSC decoder and then employ the hash for validation.

As shown in Fig. 7, during WRITE operation, we can combine the hash and baseline CHIPKILL scheme in three possible ways :

**Scheme A**: Compute the hash of data and then use SSC encoder to encode data and hash.

**Scheme B**: Encode the data and then compute the hash of encoded data.

**Scheme C**: Encode the data and compute the hash of data in parallel.

As shown in Table 5 the baseline and extended baseline provide CHIPKILL (SSC) correction capability, but with multiple symbol errors, they result up-to 8% SDC rate. The purpose of using the hash is to further reduce this SDC rate without impacting the existing reliability provided by SSC code. Therefore, while retrieving the data from the DRAM (READ operation), we use a simple, straight forward design to build upon the existing SCC capabilities. First, we perform the SSC decoding, in this process the decoder will tag each retrieved codeword to have NO Error OR Correctable Error OR Un-correctable Error. We then use the hash to validate the findings of the decoder.

On analyzing Scheme B and Scheme C with this simple retrieval mechanism we find that there is a possibility of a false positive that is, report data which was correctly handled by SSC decoder to be erroneous. This happens when the hash gets corrupted (erroneous). In this scenario, when there is a single symbol error or no error in the data/ECC symbols of a codeword, the decoder corrects it or reports that the retrieved data is free from errors, respectively. But, as the hash is corrupted in this scenario, the second step of the retrieval process reports that the data is erroneous. With Scheme A there is no scope for such false positives as hash is also correctable by SSC decoder. At the minimum, Scheme A guarantees to provide the reliability already offered by baseline (SSC decoder). In addition, it also provides capability to detect miscorrections OR undetected errors missed by the SSC decoder. Hence, we choose to pursue Scheme A further and find it to be most suitable for our purpose.

Under Scheme A, we first generate the hash of the data before encoding it with the RS-SSC encoder. This encoded data, hash pair (codeword) is stored in the memory during WRITE. When this stored codeword is retrieved from the memory during READ, we first employ the RS-SSC decoder to correct/detect errors. The RS-SSC decoder corrects up to one symbol error in each codeword to retrieve data, hash pair. As noted earlier, there is a possibility of silent data corruption in the retrieved data, hash pair if there are multiple symbol errors in the codeword. To detect this scenario, we recompute the hash of data retrieved from the SSC-RS decoder and compare it with the retrieved hash. If the hashes match, then with a high probability, we can conclude that there are no SDCs in the retrieved data. When the two hash values do not match, this indicates the presence of multiple symbol errors. Thus, we can effectively avoid silent data corruptions.

When there is up to one symbol error per codeword, this combined scheme (Scheme A, SCC decoding + Hash validation) corrects the codeword (similar to the baseline scheme) and pass on the requested 64-byte cache-line to the processor. Hence, applications waiting for this cache-line can resume their execution almost immediately on the processor. But if there is a multi-symbol error in any of the codeword, our scheme would detect that with high probability and prevent silent data corruption. This is a significant improvement over the baseline scheme.

### WRITE operation

**Figure 8:**
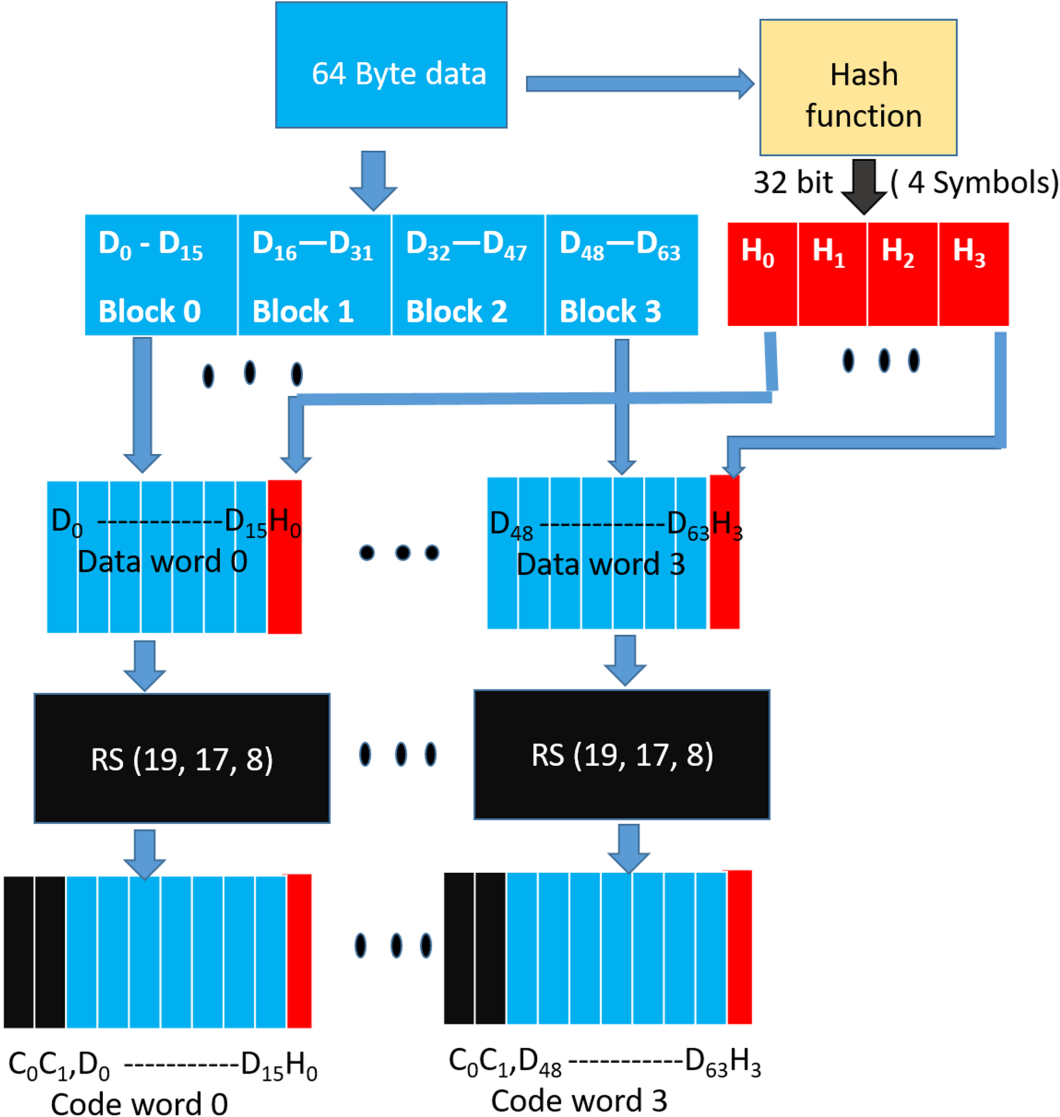
SSCMSD Design. A total of 32 bit hash is split into four symbols (red). Each hash symbol is combined with a 16-symbol data block (blue) to form a dataword.

As shown in Fig. 8, during a WRITE operation, we use a hash function to generate 32 bit output (4 symbols) from the entire cacheline (64 Bytes). Similar to the baseline SSC-RS scheme, the 64 Byte data is divided into 4 blocks (Block$_0:amp:minus;Bloc{k_3}$), each block is composed of 16 symbols. We distribute the 4-symbol hash output across the 4 data blocks by combining each data block of size 16 data symbols with 1 hash symbol to obtain a dataword. The size of our “extended” dataword is 17 symbols, as opposed to 16-symbol dataword used in the baseline design. Each dataword is encoded using RS (Jacob, Ng & Wang, 2007; Kim et al., 2012; Siddiqua et al., 2013) code to obtain a 19-symbol codeword. This 19-symbol codeword is interleaved across 2 beats as in the baseline design. Therefore, we need a total of three additional chips (storage overhead of 18.75%) per rank. We also need 12 redundant bus-lines in every channel instead of eight to accommodate the additional chip.

### Read operation

**Figure 9:**
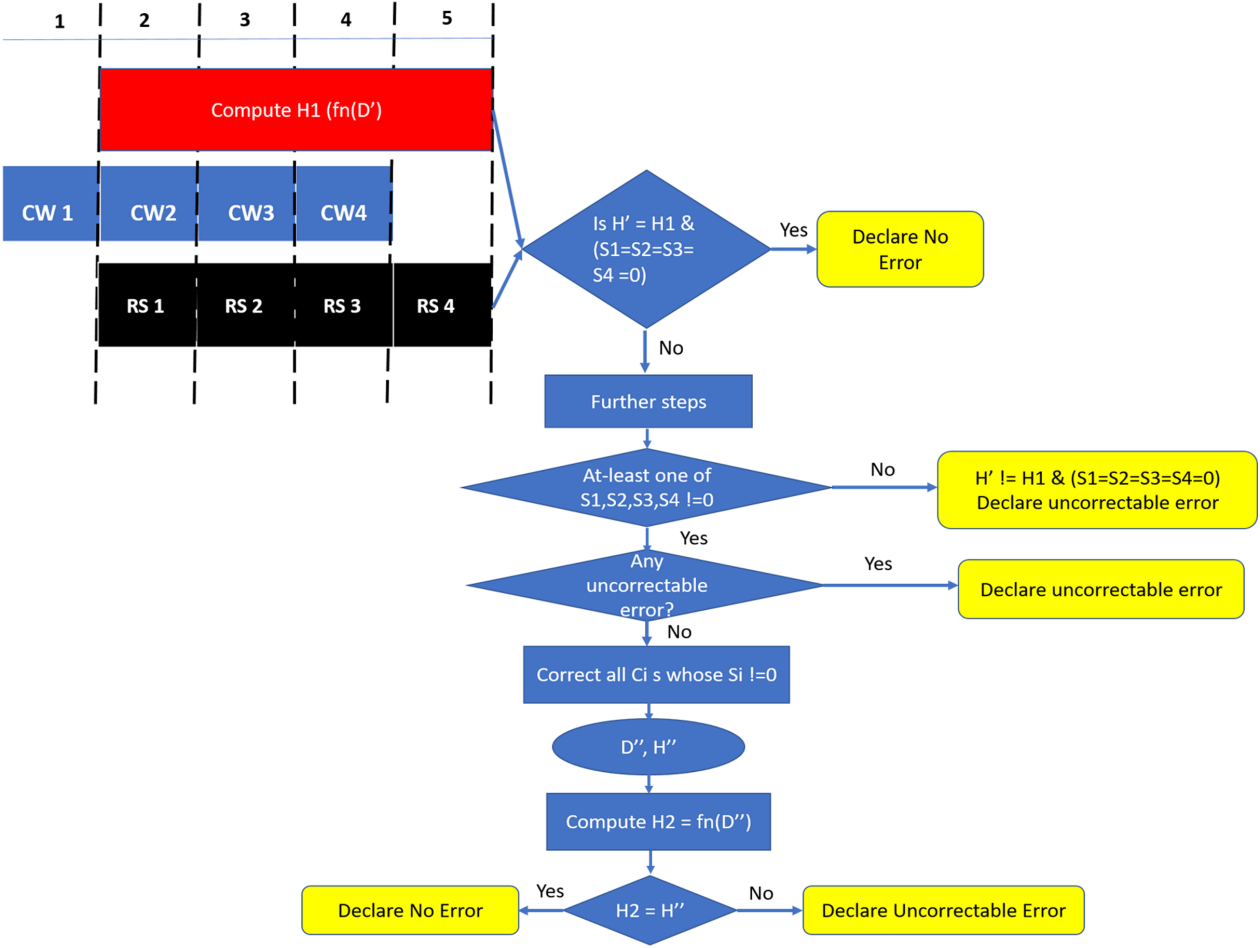
SSCMSD Design. During Read operation, syndrome computation and hash calculation are done in parallel. If any of the syndromes are non-zero AND if there are no uncorrectable errors, SSC correction is employed and Hash is computed to detect Silent Data Corruptions.

Similar to the baseline scheme, during a READ request (or MISS) two consecutive incoming data beats at the memory controller are combined to obtain a 19-symbol codeword. As shown in Fig. 9, for DDRx systems, the codewords of this READ request are obtained in four consecutive bus cycles. We need to employ SSC decoder on each codeword to obtain the 64-byte data and then validate this data with the help of hash function. As this two-step approach introduces additional latency to the READ MISS, in the following paragraphs, we describe our novel design to minimize this latency.

The SSC-Reed Solomon decoding on the received codewords is typically done in two phases. In the first phase, syndrome is computed to identify if there are any errors. Error correction (second phase) is computationally more expensive and therefore is triggered only when syndrome computation block detects errors. Since errors are relatively rare, the average delay incurred due to decoding will be close to the error free case where only the syndrome computation is performed. Study Pontarelli et al. (2015) mentions that the delay of SSC-RS syndrome calculation is about 0.48 ns with 45 nm VLSI design library. For DDR4 (Dell, 1997) with a memory clock frequency of 1,600 Mhz, syndrome computation can be implemented within one memory cycle.

The detection capability of our scheme depends on hash function properties such as length, collision resistance, avalanche behavior, distribution etc. (Estébanez et al., 2014). Also, a non-cryptographic hash (compared to cryptographic one) is sufficient for our design to limit the computation time and keep the READ latency under control. Studies Estébanez et al. (2014), Cheng & Yan (2017) show that non-cryptographic hashes—CityHash, MurmurHash, Lookup3 and SpookyHash have good properties with respect to avalanche behavior, collision resistance and even distribution. CRC-Hash is also widely used due to its simple hardware design and due to its linear property. We analyzed the hardware design of Lookup3, Spookyhash (Nelson et al., 2016; Yeleswarapu & Somani, 2018), CRC-Hash (Mytsko et al., 2016) and found that these can be implemented using combinational logic. Therefore, these hash functions can be easily implemented within four memory cycles.

Since we are using systematic SSC-Reed Solomon code, RS syndrome calculation and hash computation can be done in parallel. As shown in Fig. 9, DDRx provides two beats of data per memory clock cycle, hence SSC-RS syndrome calculation (shown in the figure as RS) and hash computation can start at the second cycle. Both these operations can be completed in five memory cycles. Each codeword received at the memory controller for decoding has 16 data symbols, one hash symbol and two ECC symbols. We denote the 64 data symbols and four hash symbols obtained from all the codewords which are not yet decoded by RS decoder as D′ and H′ respectively. We first compute the hash (H1) of the 64 data symbols (D′) and compare it with H′.

The retrieved hash H′and the computed hash H1 match if:

**Case A1**: There is no error in H′ and D′, OR

**Case A2**: D′ != D (the original 64-byte data stored/written in the memory) due to some error and H’ = H (the original hash stored/written in the memory), but due to hash aliasing H′ = H1, OR

**Case A3**: H′ != H due to some error and D′ != D due to error, but H1 (function of D′ = H′).

The retrieved hash H′ does not match H1 when there is error in hash OR 64-byte data OR in both.

In parallel, the RS decoder calculates the syndrome S_i_ for each codeword CW_i_.S_i_ can be equal to 0 when:

**Case B1**: There is no error in CW_i_ OR

**Case B2**: There is an undetected error in CW_i_.

Similarly, the syndrome is non zero when there is an error in the codeword.

Based on comparison of H1 520 and H′ and four values of *S*_*i*_ for *i* = 1 to 4, we come up with a decision table (Table 4). In the scenario where both the hashes match and syndrome is zero for all the four codewords (Scenario 1), we declare the cache-line to be free from errors. Theoretically, there is scope for silent data corruption here as it could be because of case A2 “OR” A3 “AND” case B2 for all the four codewords. From our preliminary experiments in “SSCMSD—A Novel Architectural Solution For Multi-Bit/Multiple Symbol Errors”, we notice that the probability of undetected errors (B2) very small (0.001%) for each codeword. The probability reduces further when considering the scenario of undetected errors over all the four codewords “AND” occurrence of hash aliasing (case A2) “OR” case A3. Therefore, we declare this scenario to be free from errors. For Scenarios 2 and 4 where at-least one of the syndromes S*i* is not zero, we can try to correct with the help of SSC-RS and verify again with the hash. In the scenario 3, where the hashes do not match and all S_*i*_ are 0s, we declare the cache-line to have an undetectable error due to error in data OR in both data and hash.

As the error free scenario is more common when compared to erroneous scenarios, we design our READ operation in a way that minimizes latency in the error free scenario. Therefore, as shown in Fig. 9, we check for Scenario 1 at the end of five cycles and declare the cache-line to be free of errors if Scenario 1 is found to be true. Otherwise, there are two possibilities, either at-least one of *S*_*i*_ = 0 to determine if each such codeword has a “correctable error” (CE) or “detectable-uncorrectable error” (DUE). If any one of them is an uncorrectable codeword, we declare the entire cache-line to be uncorrectable. Otherwise we correct all such codewords to obtain the corrected 64-byte data (D″) and 32-bit hash (H″). In this case, there is a scope for Silent Data Corruptions (SDCs), therefore, we compute the hash H2 from D″ and compare H2, H″. If these hashes match, then with a high probability there is no silent data corruption. If the hashes do not match, then we can conclude that SDC occurred.

Thus, we are able to reduce SDCs with our novel approach. We show later in “Tradeoff Analysis With Baseline Scheme” that on an average, the additional latency introduced per each READ miss is one memory clock cycle. Note, that this is exactly equal to latency overhead in the baseline (SSC-RS) scheme.

There is scope for false negatives (report no error although SSC decoder fails in presence of multiple symbol errors) due to hash collisions. The probability of false negative is estimated by using the upper bound on SDC rate for the SSC RS-decoder (8.0%) and aliasing probability for a N-bit hash ${(2^{ - N}})$. The upper bound on false negatives for our scheme is given by:

(7) $$Upper\,bound\,on\,P(false\,negative{) = 0.08*2^{ - 32}}$$

### Address protection

As described in “Error Model”, the byzantine address faults and MC-DIMM address faults lead to Memory data corruptions during WRITE and Silent data corruptions during READs.

The 32-bit hash we used in SSCMSD design can also be used to detect multi-bit errors in the address bus. We cannot detect errors during WRITEs as we the hash logic is available on the MC and can be employed for verification only during the READ operation. Address errors during WRITES can be tackled only by the help of logic on the DRAM device (like CAP, eWRITECRC).

**Figure 10:**
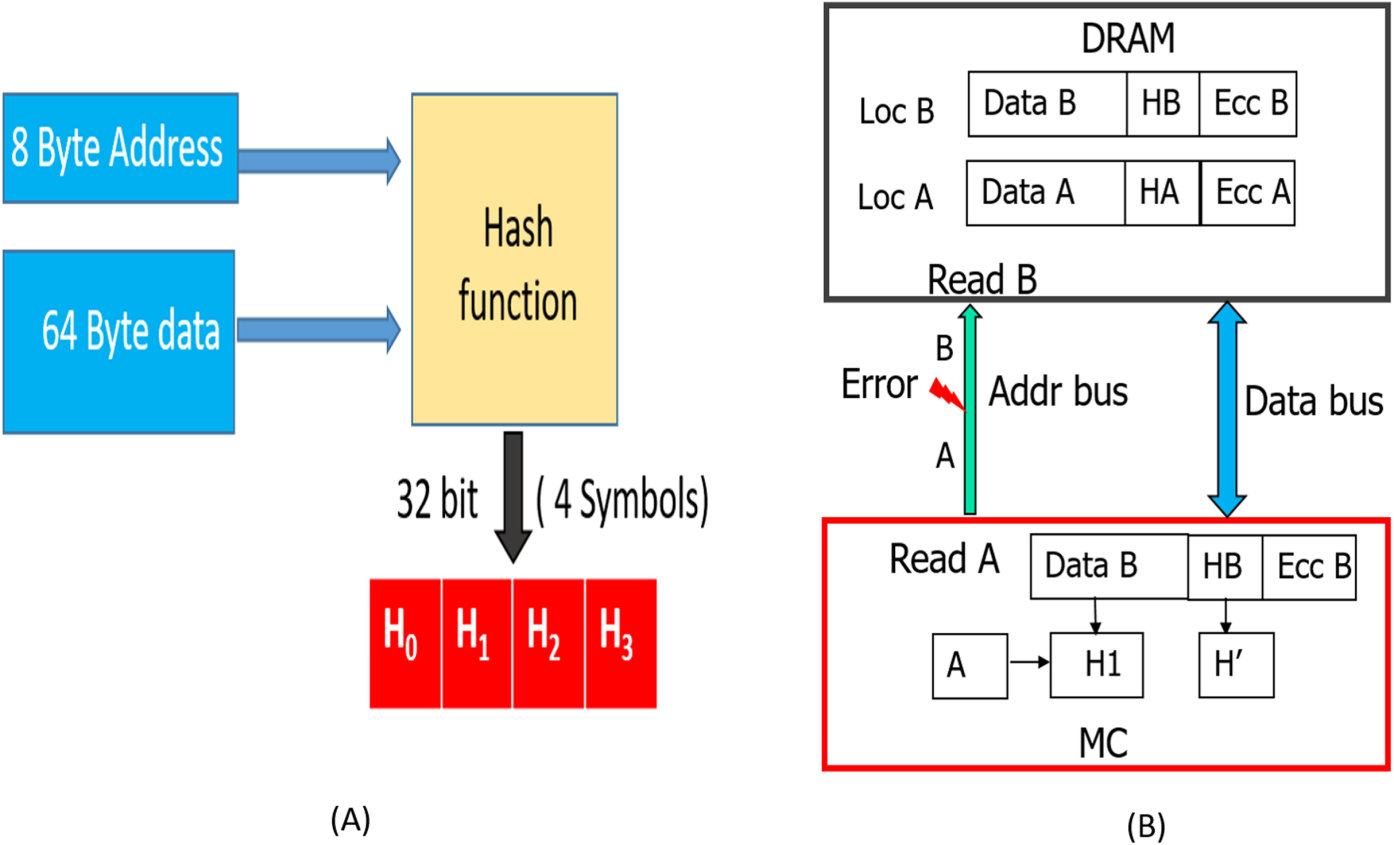
(A) Hash is now a function of both data and address. (B) Detection of MC-DIMM Address Errors during READ operation with SSCMSD.

This improves the reliability by preventing SDCs due to MC-DIMM address-errors during READs. As shown in Fig. 10A, we can hash all the address bits (8 bytes) along with the data. This hash (H), which is stored (during WRITE operation shown in Fig. 8) in the form of 4 symbols in the DRAM memory will protect against both, data and MC-DIMM address (during READs) corruption. During the READ operation, as the memory controller (MC) generates the address, it already has the correct address (address A). So, the hashes H1, H2 described in “Read Operation” will now be a function of both Data (D′/D″) and the correct address. As shown in Fig. 10B, when a transmission fault results in corruption of address (address A) bits during a particular READ request, all the DRAM devices inadvertently process it as READ request for location B. Therefore, the DRAM devices send the codewords stored at location B to the MC. At location B, we have the data “B”, hash (HB) of “data B and address B” and ecc of “data B and HB”, so the RS-decoder at MC, will not be able to detect any error. But, the hash H1 (hash of “address A and data B”) will not match with hash H′ (HB) and hence Silent data corruption is prevented.

When there is no address corruption during the prior WRITE operation (WRITE A), this design detects SDCs as expected. But, if corruption in address, during prior WRITE operation for this location/address, similar to baseline, with SSCMSD, the MC will receive stale data, hash pair (A, HA (hash of data A, address A)). The hash computed by MC will be H′ (hash of data A (stale data), address A), therefore both hashes will match, and our scheme cannot prevent Silent Data corruption. If one writes data to an unintended location due to address corruption, there is no way to detect such errors unless address is also stored along with data in DRAM. CAP (JEDEC, 2020a) and eWRITECRC (Kim et al., 2016) can take care of address corruption during WRITEs.

Therefore by including address in the hash, our scheme improves the reliability by also preventing SDCs due to MC-DIMM address-errors during READs provided there was no address corruption during the prior WRITE operation.

## Evaluation

We evaluate our scheme and compare it with the existing schemes and their extensions with the same overhead as our scheme. Baseline (RS-SSC(18,16,8)) and Bamboo-ECC (QPC Bamboo ECC (Kim, Sullivan & Erez, 2015)) use 18 chips per rank and provide CHIPKILL capability. SSCMSD uses 19 chips per rank. Therefore, we extend both the baseline and Bamboo-ECC suitably with additional redundancy to create equal overhead conditions. As described in “3 Check Symbol Based SSCDSD (Single Symbol Correct, Double Symbol Detect) CHIPKILL”, Extended Reed Solomon based (19,16,8) code uses 3 ECC symbols and provides SSCDSD (Single Symbol Correcting and Double Symbol Detecting) capability. We use this code as the extended version of the baseline. Bamboo-ECC can also be extended by using 4 more ECC symbols. This 12-ECC symbol extended version of Bamboo-ECC can be implemented in two versions. If we use all the ECC symbols for correction, then we can correct up-to 6 error-symbols. We implemented and performed simulations for this version (v1). One can also construct 8 symbol detecting and 4 symbol correcting code (v2). We did not implement and run simulations for this version, instead we provided the predicted behavior in our comparison, shown in Table 5.

The goal of our experiments is to compare the number of Silent Data corruptions across all the schemes for our error model. As described in “Error Model”, our error model covers various type of faults that arise in DRAM devices, data-bus and address bus. We classify fault modes to be causing up to one OR two OR three symbols/CW to be erroneous for the baseline, extended baseline and SSCMSD schemes. As Bamboo and extended Bamboo use vertically aligned codeword, our error model effectively translates to cause 2–12 symbols to be erroneous. For the rest of the discussion, we use the terminology of error model relative to the baseline scheme.

The following mechanisms are used to introduce errors in the encoded cacheline stored in DRAM subsystem:

1. Single bit fault: Flip a random bit in the cacheline.
2. Single pin fault: As two beats are interleaved in the baseline scheme to form one codeword, each 8-bit symbol is composed of four 2-bit pairs. As each chip has 4 data pins, each 2-bit pair of this symbol is transferred via one pin. We therefore choose a DQ pin randomly and flip two corresponding consecutive bits of a symbol.
3. Single memory chip fault/failure: Choose a chip randomly and replace the data in the chip with a random pattern OR with all 0s OR all 1s.
4. Single bus fault: Choose a bus lane randomly and use an 8-bit random number to identify the erroneous beat positions among eight beats. As each bus-lane transfers eight beats, we then inject random errors in these positions. We ensure that at-least one word of this faulty bus lane is corrupted.

A total of 1-bit, 1-pin, Row/Chip/Bank, Column, Bus faults cause errors within 1 Chip or Bus lane. Correlated bus fault affects two consecutive bus lanes. As discussed in the error model, we evaluate the following 2-chip/symbol fault modes: 1 bit fault + 1 bus fault, 1 bit fault + 1 row/bank/chip fault, 1 bit fault + 1 pin fault, 1 pin fault + 1 pin fault, and chip+chip fault. As discussed in the error model, 3 fault, multi-symbol fault (we inject 4, 5 random symbol errors) modes are also included in our evaluation.

### Simulation methodology

**Figure 11:**
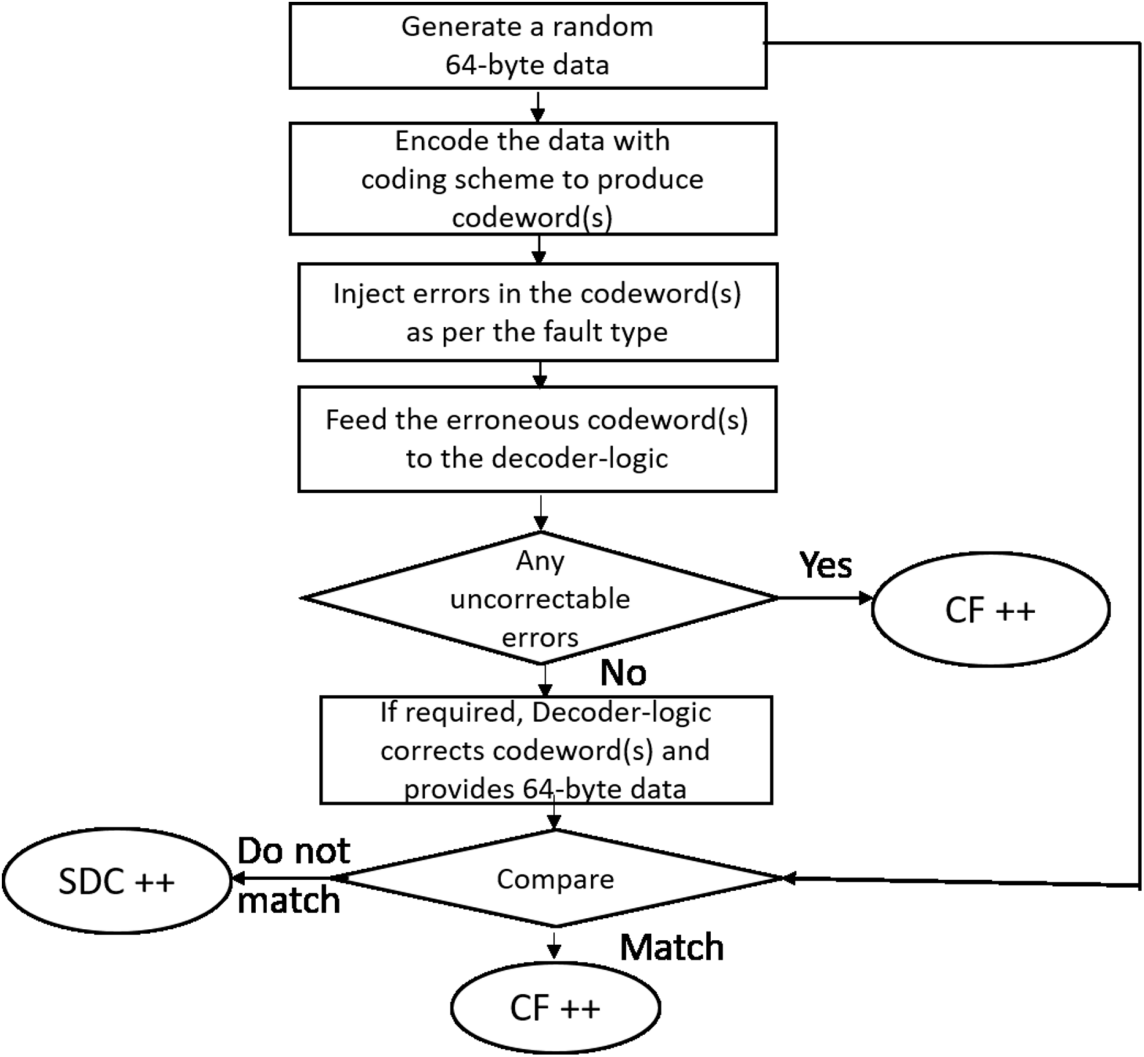
Simulation methodology.

As shown in Fig. 11 for each run, we generate a 64-byte random data (representing a cache-line). The cache-line is now encoded with the specific scheme and appropriate errors are injected as per the fault mode. The corrupted encoded cacheline is fed to the corresponding decoder logic. As described earlier, Baseline, Extended-baseline and SSCMSD use four codewords per each cache-line whereas Bamboo, Extended-Bamboo use only one codeword. Accordingly, the baseline and Extended-baseline decoder logic use four RS decoders. Bamboo and extended bamboo employ only one RS decoder in their decoder logic. For SSCMSD, we use the decoder logic described in “Read Operation” and we use random 72 bytes, each time to include 8 bytes of random address along with the cache-line. The decoder-logic will then determine whether this cache-line has Detectable Uncorrectable Error(s) (DUE) or Detectable Correctable Error(s) (DCE) or no error(s). If the logic flagged any one of the codewords of the cache-line to be a DUE, we do not suspect the decoder to be wrong as our error model has multiple symbol errors (2, 3) beyond the SSC-RS correction range. In this case, the entire cacheline has to be a DUE as this cacheline cannot be consumed and we report the whole cacheline to be a correctly flagged (CF) by the decoder. For the remaining non-DUE cachelines, we compare the original (non-corrupted) 64-byte cacheline with the cumulative output of the decoder-logic. If they do not match, we report it to be a Silent Data Corruption (SDC). Otherwise we report that the scheme (decoder) correctly flagged (CF) the cacheline.

We generate one billion runs for every iteration and execute each simulation (or experiment) for 10 iterations. Table 5 lists the mean % for these statistics across 10 iterations. The standard deviation for each of the experiments (except for SSCMSD) was up to 10,000 (for 1 billion cachelines).

### Experiments for MC-DIMM address protection

We executed simulations to verify the effectiveness of SSCMSD in the presence of address errors in MC-DIMM interface during READs. As noted in “Address Protection”, SSCMSD can provide protection against address errors during READs. So, in these simulations, for each run, we generated random 72 bytes (representing the 64-byte cacheline data and 8 byte address) and computed the hash (HA) of this “data, address” pair. Then, we used a 8-byte error mask to introduce random errors in the address bits. Next, we computed the hash (HB) of this “data, corrupted address” pair and compared HA and HB. If they differed, we declare that our scheme detected the errors (correctly flagged), otherwise we declare that there was silent data corruption. We executed this simulation for 10 iterations. Each iteration comprised of 100 billion runs. Across these 10 iterations the mean of SDCs (with CRC-32(castagnoli) as hash) was 24.5 runs with a standard deviation of 4.3. The remaining were correctly flagged (detected as errors) by our scheme.

### Comparison of SSCMSD with baseline and bamboo and their suitable extensions

As 1-bit, 1-pin, Row/Chip/Bank, Column, Bus faults result in errors confined within 1 symbol, they are corrected by all the schemes. Faults which lead to 2 or 3 symbol errors in at least one of the codeword lead to SDCs rates ranging from 0 to 7.6% in Baseline. As extended baseline uses RS (19,16,8) SSCDSD code, it can detect all 2 symbol errors. But with 3 symbol errors and multi-symbol (4,5) errors, based on our specific implementation, we observed less than 0.1 % SDCs. Both these schemes can be protected against only odd-bit errors in the MC-DIMM address bus using Command Address Parity (CAP JEDEC, 2020a). Also, as described in the “Read Operation”, the latency overhead during READ for both these schemes is 1 memory cycle.

A total of 1-bit + 1-pin fault and 1-pin + 1-pin fault modes result in two symbol errors for Bamboo-ECC and extended Bamboo-ECC (both v1 and v2), hence they are corrected by them. As extended Bamboo-ECC (v1) can correct up-to six symbol errors it can provide 100% correction with 1-bit fault + (row/chip/bank) fault and 1-bit + 1-bus fault modes. So, in the above mentioned scenarios, these schemes are better than SSCMSD (which can only detect). But with chip+chip fault and greater than 2 symbol/chip failures they have SDCs due to mis-corrections. Extended Bamboo-ECC (v2) can detect upto 8 symbol errors, therefore all 2-symbol errors will be detected. When the number of symbol errors are greater than correction capability with Bamboo and extended Bamboo (v1), the percentage of SDCs was about 11–12%. Therefore, we expect SDCs due to miscorrections to be around 10% for v2 as well. Bamboo-ECC based schemes can also be protected against odd-bit errors in the MC-DIMM address bus using Command Address Parity (CAP JEDEC, 2020a). The latency overhead (READ) for Bamboo-ECC based schemes is 4 memory cycles as the RS decoding can be done only after four memory cycles.

On the other hand, SSCMSD is able to avoid SDCs in all of the device/data fault modes. If the RS-decoder does any miscorrections, with the help of the hash we can detect and avoid SDCs. Although Bamboo-ECC, extended Bamboo-ECC (v1) can provide better correction capabilities in certain faults, with 3 and multi-symbol faults (or greater), they are prone to SDCs. The hash also provides enhanced protection in MC-DIMM address bus during READs as it can detect random errors. We are able to achieve this with a latency overhead of 1 memory cycle (same as baseline).

## Hash Selection

As described earlier, we consider non-cryptographic hash functions—SpookyHash (Spookyhash-short (Jenkins, 2012)) as our key size with data, address is 72 bytes, Lookup3 (hashlittle (Jenkins, 2006)), and CRC-32 to be employed in our SSCMSD scheme.

Minimum hamming distance (HD) and parity are important parameters useful for deciding the generating polynomial for CRC-32. For keysize of 72 bytes, CRC-32 polynomials such as Castagnoli (1,31), koopman32k (1,3,28), koopman32k_2_ (1,1,30) provide minimum HD of 6 (Koopman, 2002). Therefore, errors up-to 5 (= 6 − 1) random bit flips are guaranteed to be detected by these polynomials. Also, the above mentioned HD = 6 polynomials have even parity, hence they guarantee detection of all odd bit errors. IEEE 802.3 (32) polynomial provides a minimum HD of 5 for our keysize and has odd parity.

In Table 6, we compare the properties and performance (SDC detection capability) of SpookyHash-short, Lookup3, CRC-32-Castagnoli (as a representative of HD = 6, even parity polynomials) and CRC-32-IEEE 802.3 (as a representative of odd parity polynomial) hash functions. The goal of our experiments is to identify the one which is most suited to be with SSCMSD.

**Table 6:** Performance of different Hash functions with SSCMSD design.

Hash used with SSCMSD	SDCs in presence of address faults (100 billion runs)	Linear property	Other properties
Spookyhash short (32-bit)	up to 25	No	–
Lookup3 (hashlittle)	up to 27	No	–
CRC-32 (castagnoli)	up to 27	Yes	HD = 6, even parity
CRC-32 (IEEE 802.3)	up to 30	Yes	HD = 5, odd parity

We executed all the data fault simulations described in Table 5 for 10 iterations with 1 billion runs per iteration and 10 billion runs per iteration for these hash functions. We also executed random address fault simulations described in “Experiments for MC-DIMM Address Protection” for 10 iterations with 10 billion runs per iteration and 100 billion runs per iteration. Note that we executed address fault experiments with 100 billion runs/iteration as error detection in this case is exclusively dependent on the hash function (whereas for data errors, both the RS decoding and hash work together to avoid SDCs) and as experiments did not involve RS encoding and decoding, we were able to run for a larger number (compared to 1 billion, 10 billion runs for data faults) of runs in a reasonable amount of time.

For all the data fault experiments (both 1 billion and 10 billion runs/iteration) and address fault experiments with 10 billion runs/iteration, we did not observe any SDCs for all the four hashes (these results are therefore not included in Table 6). We do not observe any SDCs due to hash collisions in these simulations due to the nature of SSCMSD (or baseline) design. As described in Fig. 9, SSCMSD uses four codewords and the hash is employed only when all of four codewords are corrected (none of them are DUEs) by the RS-SSC (19,17,8) decoder. Due to the sparsity of RS (19,17,8) SSC space, the probability of anyone of the four codewords to be a DUE is around 90 percent and therefore, only 10 percent of the time the hash is employed. For the address fault experiments with 100 billion runs/iteration, we observed upto 30 SDCs per iteration as listed.

Since we did not observe any significant differences among the different 32-bit hash functions we considered, we can employ any one of HD = 6 CRC-32 polynomials (Castagnoli, or koopman32k, or koopman32k_2_) with our SSCMSD design as they are simple, provide minimum HD = 6 codewords with even parity and enable us to compute the hash in parts (due to linear property) during the READ operation.

## Tradeoff Analysis with Baseline Scheme

In this section, we compare the tradeoffs/costs of SSCMSD’s design with the baseline (AMD’s SSC-CHIPKILL) scheme. Table 7 compares reliability, redundancy, area/logic, latency and power of both the schemes. As described earlier, the baseline makes use of SSC-RS codes and requires two additional devices per DRAM rank (storage overhead of 12.5%). SSCMSD judiciously combines hash (CRC-32 of data and address) with SSC-RS codes and requires three redundant devices per DRAM rank (storage overhead of 18.75%). As the number of data bus-lanes/lines per channel increase in proportion to the number of devices on each rank, baseline uses a total of 72 bus-lines (two additional bus-lanes) and SSCMSD needs 76 bus-lines (three additional bus-lanes). SSCMSD does not introduce any additional command/address lines.

**Table 7:** Tradeoffs analysis with baseline scheme.

Criteria	Baseline	SSCMSD
Storage Overhead	12.5% (2/16)	18.75% (3/16)
Data bus width	72	76
Single symbol data error	100% correction	100% correction
Multiple symbol data errors	SDCs up to 7.6%	no SDCs, false prob ${0.08*2^{ - 32}}$
Address errors READs WRITEs	Detects odd # errors odd # errors	Detects random errors odd # errors
Fraction of memory power (fmp)	25–46%	26–47.3%
Latency during READs WRITEs	+1 mem cycle +1 mem cycle	+1 mem cycle +2 mem cycles
MC Logic	RS logic	RS + CRC-logic

Both baseline and SSCMSD guarantee correction up to one symbol error/CW in the data. As shown in our evaluation, baseline incurs upto 7.6% SDCs with multiple symbol errors in data whereas SSCMSD detects them with a very high probability (upper bound on false negative probability is 0.08 ∗ 2^−32^). SSCMSD detects random MC-DIMM address errors during READs with a high probability (false negative probability is 2^−32^) without introducing additional address pins. On the other hand, using Command Address Parity (CAP JEDEC, 2020a), only odd-bit errors are detected in the baseline during READs. During WRITEs, both schemes make use of CAP to provide limited detection capability, hence can only detect odd-bit errors.

The power consumed by a DRAM device is dependent on the mode/state of operation of the device (Micron Inc., 2017). Memory system power (sum total of power consumed by all devices and associated bus-lines) in a cluster/HPC is also dependent on system configuration such as capacity, number of channels per socket, number of DIMMS per channel, number of ranks per DIMMs, frequency of operation etc (Gough, Steiner & Saunders, 2015).

As SSCMSD/AMD-CHIPKILL schemes are typically employed in cluster/HPC environment, we are interested in the fraction of memory power to the overall system power (fmp). Note that system power of a server/cluster comprises of contributions from compute, memory (DRAM), I/O, Interconnect chips, Storage, Cooling (Bose, 2012). The total system power can be broken into two components: memory system power (Msys) and contribution from the remaining components (Rsys). Therefore, fmp can be expressed as:

(8) $$fmp = \displaystyle{{Msys} \over {Msys + Rsys}}$$

With 18 devices per rank, the fmp for cluster/system using baseline scheme is given by Eq. (8). As SSCMSD uses 19 devices per rank (and 19 bus-lanes), if we assume that the other factors remain the same (Rsys remains unchanged), the memory system power (Msys) of a similar system with SSCMSD will be 19/18 times the original value and the fmp for such a system is given by Eq. (9).

(9) $$fmp - {\rm SSCMSD} = \displaystyle{{(19/18)*Msys} \over {(19/18)*Msys + Rsys}}$$

To get a sense of the power overhead, we use the information presented in recent studies (Ghose et al., 2018; Bose, 2012; Malladi et al., 2012) which show that fmp is about 25–46%. We assign this fmp for a system using baseline scheme, from Eqs. (8) and (9), the corresponding fmp of system using SSCMSD (fmp-SSCMSD) turns out to be between 26 and 47.3%.

As described earlier, the baseline makes use of RS (18,16,8) block (RS encoder, syndrome and correction logic) at the memory controller. The study Pontarelli et al. (2015) designed this particular RS-block (referred in (Pontarelli et al., 2015) as SEC-RS(18,16,8)) with 45 nm technology (FreePDK45 (Stine et al., 2007) library), and provided the area, delay information. We use this information to compare the designs employed by baseline and SSCMSD at the memory controller.

The study Pontarelli et al. (2015) mentions that the delay of encoder and syndrome of this RS-block is 0.47 ns and 0.48 ns respectively with FreePDK45 (Stine et al., 2007). Their design allowed the RS-block to operate at the maximum clock frequency, given by the inverse of syndrome delay (2,083 Mhz = 1/0.48). Hence, this block can be easily executed with memory clock frequency of 1600 Mhz (highest I/O frequency of DDR4 (Dell, 1997)) at the memory controller (MC). To summarize, this RS block would require 1 memory cycle for encoding and 1 memory cycle for syndrome computation at the MC. As described in “Read Operation”, the average delay incurred during READs will be approximately equal to delay associated with syndrome computation. Therefore, the baseline scheme will have +1 memory clock penalty during READs (due to syndrome computation) and +1 memory clock penalty (due to RS encoding) during WRITE operation.

SSCMSD needs CRC logic along with the RS block at the memory controller. We have synthesized a fully parallel and combinational CRC-32 (CRC-Castagnoli (1,31)) logic using RTL code based on (Stavinov, 2010) and FreePDK45 (Stine et al., 2007) (the same library used by Pontarelli et al. (2015)). This block has a delay of 0.52 ns and we were able to synthesize it easily with the clock constrained to run at 1,600 Mhz. Therefore, as expected, CRC-computation will be completed in 1 memory clock-cycle.

As described in “SSCMSD—A Novel Architectural Solution for Multi-Bit/Multiple Symbol Errors”, SSCMSD computes CRC and syndrome in parallel during the READ operation, and performs CRC-computation before RS encoding during the WRITE operation. Therefore, SSCMSD would incur +1 memory cycle penalty during READs and +2 (+1 for CRC computation and +1 RS encoding) memory cycles penalty during WRITEs. Overall, the impact of SSCMSD on application performance will be negligible as WRITEs are not on the critical path and as there is no additional overhead in the latency during READs.

The total area (encoder, syndrome and correction logic) of RS block, reported by Pontarelli et al. (2015) is 24,793 square micrometers. On the other hand, the CRC block we synthesized has an area of 5,970 square micrometers. Therefore, the impact of our scheme on area at the memory controller (MC) is also negligible.

## Future Work

One major criticism of our work could be that we add an extra part in the memory subsystem design. This chip level overhead can be traded by storing more bits in the chips and by increasing the burst length per cacheline to retrieve/transfer these additional bits. We believe that the hash can also be employed without increasing the number of devices per rank. To provide SSCMSD to a ×4/×8 based DDR4 system using storage overhead of 12.5% (same as in SSC-CHIPKILL), we can make use of additional beats to store the hash in the same chips (with more bits in each rank). Currently DDR4 supports 8 beats per each cacheline. The hash can be transferred on an additional beat (9th beat) and can be stored across all the 18 devices or 9 devices (as in LOT-ECC (Udipi et al., 2012)) for ×4/×8 respectively.

For DDR5 JEDEC, 2020b, the data bus-width per channel is 32 bits. To store 64 bytes, 16 beats are required per each cacheline. To provide CHIPKILL capability, two more devices (for ×4 DDR5) are added per channel. Here too, one can employ the hash along with ECC to provide SSCMSD. The hash can be transferred on an additional beat (17th beat) and can be stored across all the 10 (8 + 2 ECC) devices.

These schemes need to be investigated further to evaluate the performance, reliability and other impacts to make the right design choices.

## Conclusion

We motivate the need for addressing multiple symbol errors in CHIKPILL based DRAM subsystems given the trend of increase in failures in these systems. Based on the nature of these failures, we analyzed possible errors and then developed a new error-handling scheme called Single Symbol Correction, Multi Symbol Detection (SSCMSD). SSCMSD effectively guards the data in the memory subsystem from memory device, data and address bus errors.

We implemented SSCMSD using CRC-32 and Single symbol correcting reed solomon (SSC-RS) code. By leveraging the usage of systematic SSC-RS code and simple CRC-32 hash, our novel design’s impact on the READ latency is negligible. Our simulations compare SSCMSD scheme with baseline (SSC-RS) and Bamboo-ECC. The results clearly demonstrate that SSCMSD is effective in avoiding Silent Data Corruptions (SDCs) in the presence of multiple symbol errors in both data and address.

## Supplemental Information

10.7717/peerj-cs.359/supp-1 Supplemental Information 1 Code and executable for 3 Symbol Errors/CW experiment shown in Table 1 (Results of Random multi-symbol data errors for RS(18,16,8)).

## Additional Information and Declarations

### Data Availability

The following information was supplied regarding data availability: Code is available in the Supplemental Files.
