# Supplementary material for: Addressing multiple bit/symbol errors in DRAM subsystem
Source: PeerJ Comput Sci. 2021 Feb 9;7:e359. doi: 10.7717/peerj-cs.359 (PMC7959593; doi:10.7717/peerj-cs.359)
Supplement: Supplemental Information 1 [file peerj-cs-07-359-s001.zip › upload/rscode-1.3.tgz!/rscode-1.3/config.doc]

The basic coding parameters are defined using

macros, and an executable can be made by compiling using macro

definitions defining the values of the following names in the file

"ecc.h":

The important compile time parameter is the number of parity bytes,

specified by the #define NPAR.

The library is shipped with

#define NPAR 4

The error-correction routines are polynomial in the number of

parity bytes, so try to keep NPAR small for high performance.

Remember, the sum of the message length (in bytes) plus parity bytes

must be less than or equal to 255.
