# Supplementary material for: Addressing multiple bit/symbol errors in DRAM subsystem
Source: PeerJ Comput Sci. 2021 Feb 9;7:e359. doi: 10.7717/peerj-cs.359 (PMC7959593; doi:10.7717/peerj-cs.359)
Supplement: Supplemental Information 1 [file peerj-cs-07-359-s001.zip › upload/rscode-1.3.tgz!/rscode-1.3/rs.doc]

Introduction to Reed Solomon Codes:

Henry Minsky, Universal Access Inc.

hqm@alum.mit.edu

[For details see Cain, Clark, "Error-Correction Coding For Digital

Communications", pp. 205.] The Reed-Solomon Code is an algebraic code

belonging to the class of BCH (Bose-Chaudry-Hocquehen) multiple burst

correcting cyclic codes. The Reed Solomon code operates on bytes of

fixed length.

Given m parity bytes, a Reed-Solomon code can correct up to m byte

errors in known positions (erasures), or detect and correct up to m/2

byte errors in unknown positions.

This is an implementation of a Reed-Solomon code with 8 bit bytes, and

a configurable number of parity bytes. The maximum sequence length

(codeword) that can be generated is 255 bytes, including parity bytes.

In practice, shorter sequences are used.

ENCODING: The basic principle of encoding is to find the remainder of

the message divided by a generator polynomial G(x). The encoder works

by simulating a Linear Feedback Shift Register with degree equal to

G(x), and feedback taps with the coefficents of the generating

polynomial of the code.

The rs.c file contains an algorithm to generate the encoder polynomial

for any number of bytes of parity, configurable as the NPAR constant

in the file ecc.h.

For this RS code, G(x) = (x-a^1)(x-a^2)(x-a^3)(x-a^4)...(x-a^NPAR)

where 'a' is a primitive element of the Galois Field GF(256) (== 2).

DECODING

The decoder generates four syndrome bytes, which will be all zero if

the message has no errors. If there are errors, the location and value

of the errors can be determined in a number of ways.

Computing the syndromes is easily done as a sum of products, see pp.

179 [Rhee 89].

Fundamentally, the syndome bytes form four simultaneous equations

which can be solved to find the error locations. Once error locations

are known, the syndrome bytes can be used to find the value of the

errors, and they can thus be corrected.

A simplified solution for locating and correcting single errors is

given in Cain and Clark, Ch. 5.

The more general error-location algorithm is the Berlekamp-Massey

algorithm, which will locate up to four errors, by iteratively solving

for the error-locator polynomial. The Modified Berlekamp Massey

algorithm takes as initial conditions any known suspicious bytes

(erasure flags) which you may have (such as might be flagged by

a laser demodulator, or deduced from a failure in a cross-interleaved

block code row or column).

Once the location of errors is known, error correction is done using

the error-evaluator polynomial.

APPLICATION IDEAS

As an example application, this library could be used to implement the

Compact Disc standard of 24 data bytes and 4 parity bytes. A RS code

with 24 data bytes and 4 parity bytes is referred to as a (28,24) RS

code. A (n, k) RS code is said to have efficiency k/n. This first

(28,24) coding is called the C2 or level 2 encoding, because in a

doubly encoded scheme, the codewords are decoded at the second

decoding step.

In following the approach used by Compact Disc digital audio, the 28

byte C2 codewords are four way interleaved and then the interleaved

data is encoded again with a (32,28) RS code. The is the C1 encoding

stage. This produces what is known as a "product code", and has

excellent error correction capability due to the imposition of

two-dimensional structure on the parity checks. The interleave helps

to insure against the case that a multibyte burst error wipes out more

than two bytes in each codeword. The cross-correction capability of

the product code can provide backup if in fact there are more than 2

uncorrectable errors in a block.
